# *SNORD18B*/*SNORD18C*-RPL23A promotes homologous recombination repair by regulating *BRCA1* translation in colorectal cancer cells

**DOI:** 10.1038/s41420-026-03289-3

**Published:** 2026-08-06

**Authors:** Chuxian Lin, Junyan He, Lei Wu, Xin Liu, Ying Fan, Dan Cai, Meng Jia, Wencheng Zhang, Qi Wang, Yaqiong Li, Ruixue Liu, Wenqing Lu, Jianan Ma, MaoXiang Zhu, Pingkun Zhou, Zhenhua Qi, Liping Shen, Zhidong Wang

**Affiliations:** 1 Beijing Institute of Radiation Medicine, Beijing, China; 2 Department of Oncology, The First Affiliated Hospital, Hengyang Medical School, University of South China, Hengyang, China; 3 Graduate Collaborative Training Base of Academy of Military Sciences, Hengyang Medical School, University of South China, Hengyang, China

**Keywords:** Double-strand DNA breaks, Non-coding RNAs, Homologous recombination

## Abstract

Homologous recombination (HR) is one of the classical pathways for repairing DNA double-strand breaks (DSBs), a type of lesion that poses a severe threat to genomic stability and cell survival. The role of small nucleolar RNAs (snoRNAs), particularly their interactions with ribosomal proteins, in the HR repair has not been reported. This is the first study to report the role of snoRNA genes *SNORD18B* and *SNORD18C* (*SNORD18B/C*) in HR repair, demonstrating that they exert an oncogenic function in colorectal cancer (CRC) by promoting cell proliferation and radioresistance. Mechanistically, *SNORD18B/C* directly interacts with the ribosomal protein RPL23A to enhance the translation of BRCA1 independent of the canonical 2′-O-methylation of *28S* rRNA, thereby promoting HR efficiency to facilitate radiation-induced DSB repair. Collectively, our findings confirm that the *SNORD18B/C*-RPL23A-BRCA1 axis is crucial for HR repair and highlight the *SNORD18B/C* as potential CRC therapeutic targets.

**Figure:**
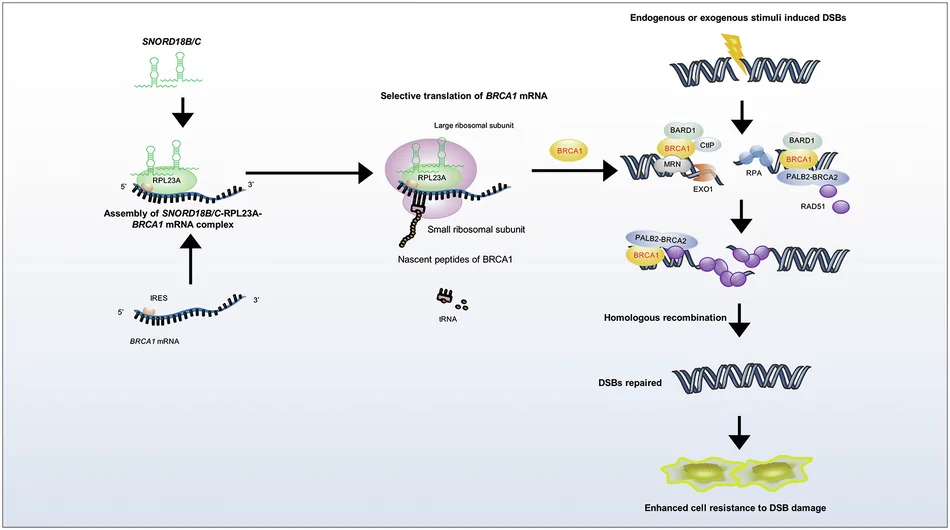

## Introduction

As a primary source of genomic instability, DNA double-strand breaks (DSBs) are continually induced by both endogenous factors (e.g., DNA replication and oxidative stress) and exogenous factors (e.g., ionizing radiation and chemical agents). The two major DSB repair pathways, non-homologous end joining (NHEJ) and homologous recombination (HR), operate under distinct mechanisms. NHEJ functions as a rapid yet error-prone method sustaining activity across all cell cycle phases. In contrast, HR repair is restricted to S- and G2-phase cells, using sister chromatid sequences as templates for high-fidelity repair [1]. DSB formed by DNA replication fork stalling or collapse, as well as clustered DSB caused by ionizing radiation, are believed to be primarily repaired by HR [2–5]. HR repair initiates with DSB end resection, a step mediated by the MRE11-RAD50-NBS1 (the yeast homolog of NBS1 is Xrs2) complex, CtIP, and several cofactors, leading to the formation of 3′ single-stranded DNA (ssDNA) overhangs [6, 7]. Replication protein A (RPA) protects ssDNA from degradation through high-affinity binding, and RAD51 recombinase then replaces RPA to guide DNA strand invasion and initiate DNA synthesis [6, 7]. The breast cancer type 1 susceptibility protein (BRCA1), encoded by the *BRCA1* gene, has been confirmed as an essential factor in HR repair— specifically during DSB end resection and RAD51-mediated strand invasion [8–10]. On the one hand, BRCA1 antagonizes 53BP1 by competitively binding to the DSB ends, enhancing HR repair via MRN/X-CtIP-mediated resection [7, 10, 11]. On the other hand, the BRCA1-BARD1 heterodimer facilitates ssDNA tail generation to enhance RAD51 recruitment, thereby augmenting strand invasion [9]. Given BRCA1’s essential role in the process of HR, it has now been utilized as both a diagnostic biomarker and a crucial therapeutic target for certain cancers [12, 13].

Small nucleolar RNAs (snoRNAs) are a class of small noncoding RNAs (60–300 nucleotides in length) predominantly located in the nucleolus. Based on their structural motifs, snoRNAs are classified into C/D box and H/ACA box snoRNAs. C/D box snoRNAs are characterized by conserved terminal motifs: a C box (RUGAUGA, R represents A or G) and a D box (CUGA), along with less conserved C′ and D′ boxes. H/ACA box snoRNAs contain a conserved H box (ANANNA, N represents any nucleotide) and an ACA/AUA box motif. SnoRNAs predominantly guide rRNA modifications, including 2′-O-methylation (2′-O-Me) and pseudouridylation, to regulate ribosome assembly [14]. Recent advances indicate that specific snoRNAs are involved in DNA damage repair [15]. For example, *SNORA73* modulates genome stability in acute myeloid leukemia cells by directly inhibiting PARP1 auto-PARylation [16]. *ScaRNA2* interacts with DNA-PKcs and inhibits its autophosphorylation to attenuate NHEJ repair [17]. However, the role of snoRNAs in regulating BRCA1-mediated HR repair has yet to be explored.

Ribosome assembly and mRNA translation are two intrinsically coupled biological processes precisely controlled by snoRNAs and highly sensitive to diverse stimuli [18, 19]. Ribosomal proteins, as the core components of ribosomes, have also been found to play critical roles in DNA damage repair [20–23]. In 2024, Cheng et al. [24] demonstrated that *SNORA13* directly interacts with RPL23, triggering ribosomal stress and p53-mediated cellular senescence, which suggests that snoRNA-ribosomal protein interactions serve a critical function in stress responses. To date, the impact of snoRNA–ribosomal protein interactions on DNA repair remains completely unknown. Ribosomal protein L23a (RPL23A), a ribosomal protein located in the 60S subunit’s exit tunnel, plays an essential role in translational regulation by facilitating the folding and translocation of nascent polypeptides [25, 26]. Although studies have reported that RPL23A regulates cell proliferation, apoptosis, and radiosensitivity in mouse spermatogonia [27], its role in HR repair has never been elucidated. This study revealed that C/D box snoRNAs *SNORD18B* and *SNORD18C* (*SNORD18B/C*) directly bind to RPL23A, leading to enhanced translation of BRCA1 protein, thereby improving HR repair efficiency, promoting proliferation, and radioresistance in CRC cells. Together, these findings provide novel mechanistic insights into snoRNA-mediated regulation of the HR repair pathway.

## Results

### *SNORD18B/C* enhances HR repair in CRC cells

Our previous studies (unpublished data) identified a subset of radiation-responsive snoRNAs (fold change ≥1.3) in peripheral lymphocytes of BALB/c mice exposed to ^60^Co γ-ray radiation. Through intersection analysis of radiation-responsive snoRNAs and those differentially expressed in CRC tissues [28, 29], we found that radiation-responsive snoRNAs *SNORD18B* and *SNORD18C* were significantly upregulated in CRC tissues (Fig. S1A and S1B). *SNORD18B* and *SNORD18C* are homologous genes (86% similarity, Fig. S1C) located in the intronic region of the host *RPL4* gene on human chromosome 15 (Fig. S1D). The sequence similarity between *SNORD18B* and *SNORD18C* implies that these genes share similar biological mechanisms or functional roles.

**Fig. 1:**
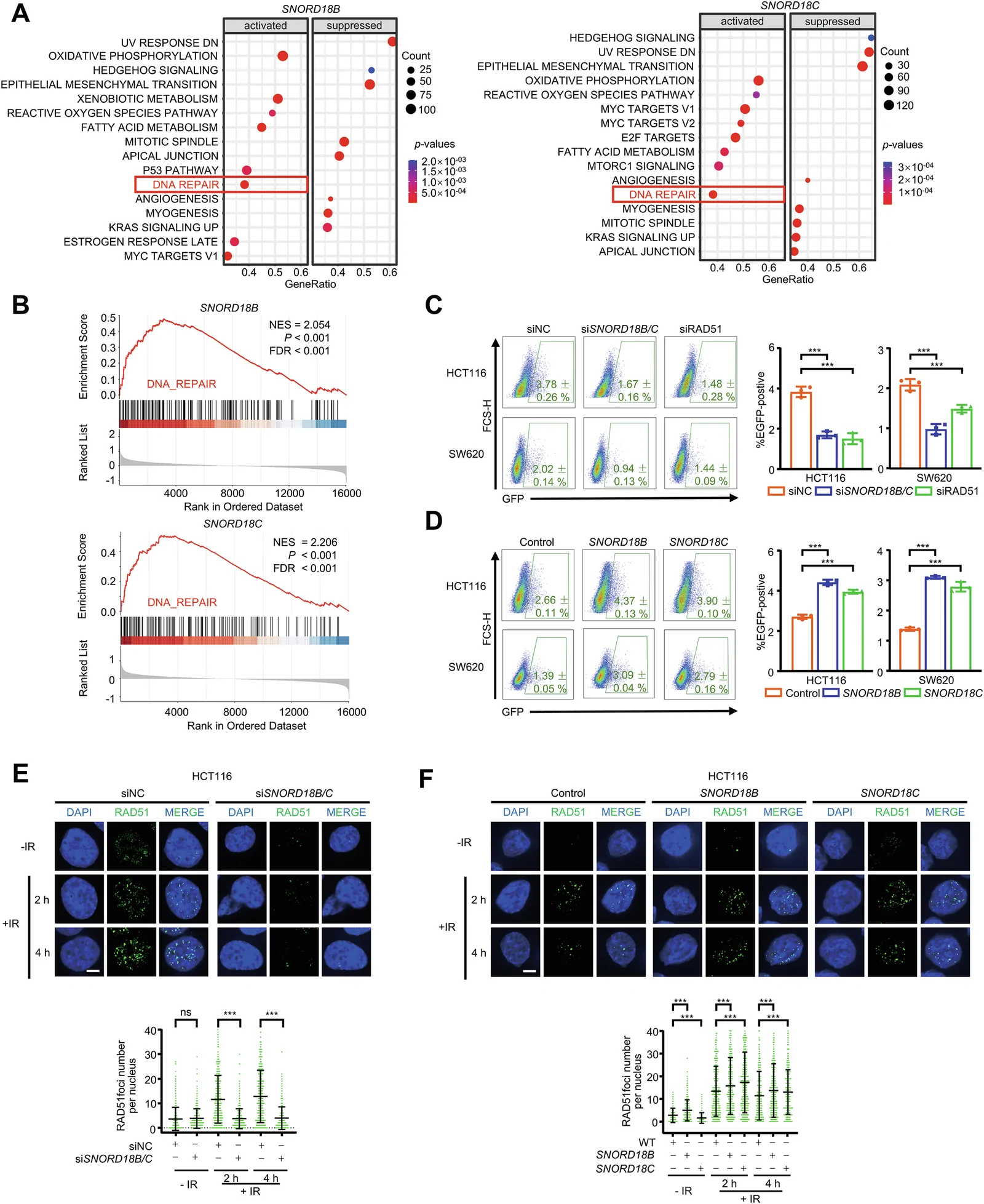
*SNORD18B/C* enhances HR repair in CRC cells. **A** Enrichment analysis of Hallmark pathways comparing patients with low and high *SNORD18B/C* expression levels. The data of the CRC patient cohorts were downloaded from the TCGA. Bubble size: enriched gene numbers; bubble color: *P* value indicating enrichment significance. **B** GSEA of TCGA CRC patient cohorts (low *SNORD18B/C* patients versus high *SNORD18B/C* patients; median cutoff value, *n* = 590). NES normalized enrichment score, FDR false discovery rate. **C**, **D** HR reporter assays. HCT116 and SW620 cell lines with *SNORD18B/C* knockdown (**C**) and overexpression (**D**) were co-transfected with DR-GFP and I-SceI plasmids, and the proportion of GFP^+^ cells was measured at 48 h via flow cytometry (mean ± SD, two-tailed Student’s *t* test, *n* = 3). **E**, **F** Immunofluorescence analysis of RAD51 was performed in HCT116 cells with *SNORD18B/C* knockdown (**E**) or overexpression (**F**) post-irradiation (at 2 and 4 h post 10 Gy radiation). Scale bar: 5 µm. The foci number in at least 200 nuclei in each group was determined via Fiji software (Mann–Whitney test). ***P < 0.001, *ns* means no significance.

To predict the function of *SNORD18B/C*, we divided CRC cohorts from the Cancer Genome Atlas Program (TCGA) database into high- and low-expression groups based on the median expression level of *SNORD18B/C* as the cutoff threshold. Hallmark pathway enrichment and gene set enrichment analysis (GSEA) revealed that the DNA damage repair pathway was significantly activated in high *SNORD18B/C* expression groups (Fig. 1A, B). To investigate the impact of *SNORD18B/C* on the DNA repair pathway, we analyzed the effects of *SNORD18B/C* on the efficiency of HR and NHEJ in HCT116 and SW620 cells. DR-GFP reporter assay revealed that *SNORD18B/C* knockdown through siRNA transfection (targeting both genes simultaneously due to their high homology) significantly inhibited HR repair, as evidenced by reduced GFP^+^ cell populations (HCT116: si*SNORD18B/C* 1.67 ± 0.16%, siRAD51 1.48 ± 0.28%, siNC 3.78 ± 0.26%; SW620: si*SNORD18B/C* 0.94 ± 0.13%, siRAD51 1.44 ± 0.09%, siNC 2.02 ± 0.14%, siRAD51 as a control). In contrast, *SNORD18B/C* overexpression in the cells via lentivirus significantly enhanced HR repair (HCT116: *SNORD18B*, 4.37 ± 0.13%; *SNORD18C*, 3.90 ± 0.10%; control, 2.66 ± 0.11%; SW620: *SNORD18B*, 3.09 ± 0.04%; *SNORD18C*, 2.79 ± 0.16%; control, 1.39 ± 0.05%) (Figs. 1C, D and S2A–C). In this study, we observed that NHEJ repair increased slightly in *SNORD18B/C*-knockdown cells with suppressed HR efficiency (Fig. S3A, B). This phenomenon, termed the “seesaw effect”, may be attributed to a compensatory selection mechanism between the NHEJ and HR repair pathways. During the process of HR repair, RAD51 foci formation serves as a reliable hallmark for assessing HR repair activity [30, 31]. To further validate the impact of *SNORD18B/C* on HR repair, we examined RAD51 foci formation in *SNORD18B/C*-knockdown CRC cells after exposure to 10 Gy ^60^Co γ-rays. We found that suppressing *SNORD18B/C* expression significantly reduced the number of RAD51 foci. Conversely, overexpression of *SNORD18B* or *SNORD18C* increased the formation of RAD51 foci, further confirming the positive regulatory role of *SNORD18B/C* in HR repair (Figs. 1E, F and S4). Current evidence suggests that the expression of snoRNAs in eukaryotes often interacts with and influences their host genes [14, 32, 33]. By interfering with *SNORD18B/C* expression in vitro, we found that the RPL4 protein (encoded by the host gene *RPL4*) remained unchanged (Fig. S5), indicating that the role of *SNORD18B/C* in HR repair is independent of its host gene expression.

### *SNORD18B/C* is involved in HR repair by regulating BRCA1 expression

**Fig. 2:**
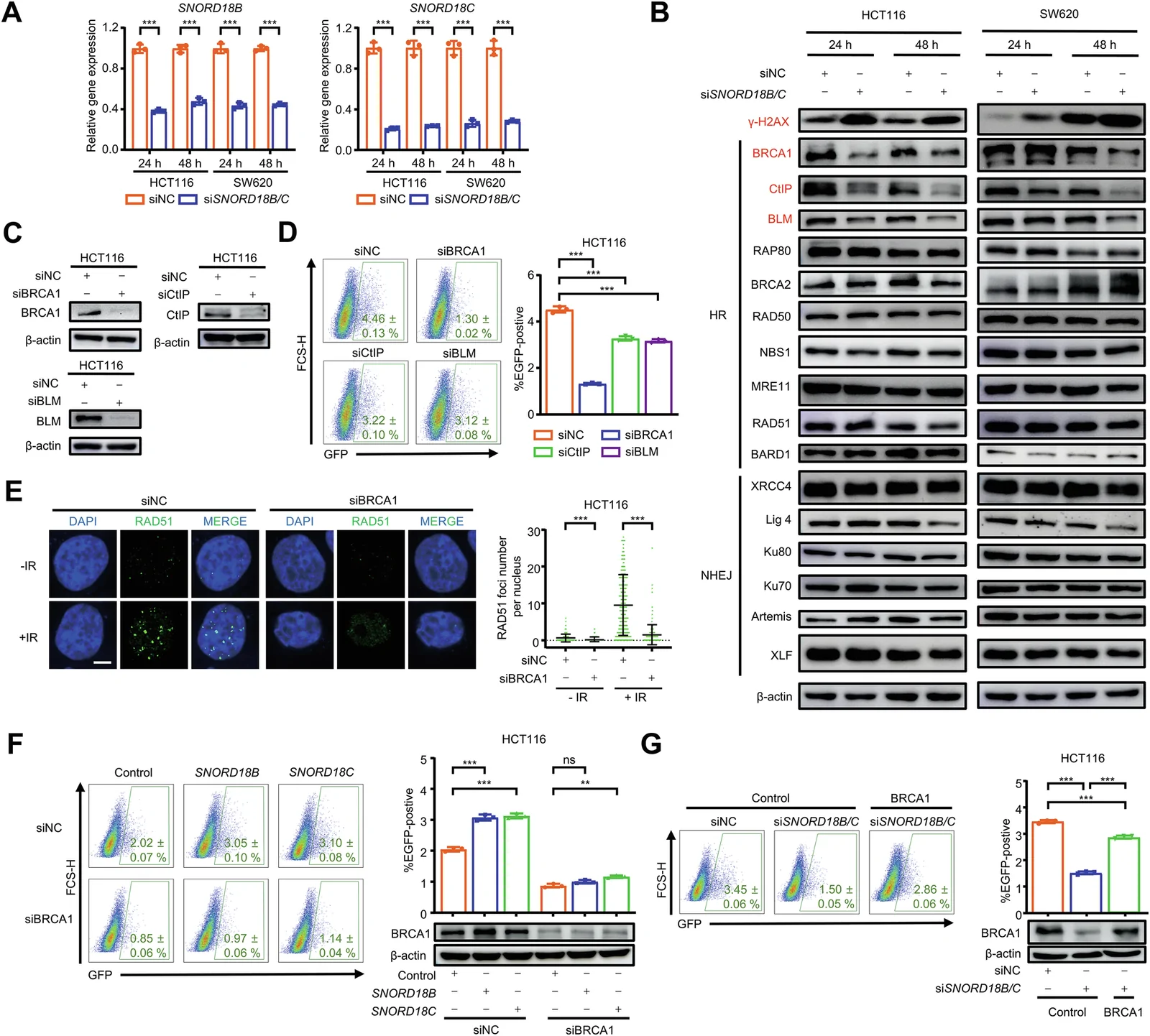
*SNORD18B/C* is involved in HR repair by regulating BRCA1 expression. **A** Analysis of *SNORD18B/C* expression in HCT116 and SW620 cells with *SNORD18B/C* knockdown. qRT-PCR was performed on HCT116 and SW620 cells at 24 and 48 h post-siRNA transfection. **B** Analysis of HR and NHEJ repair factors in HCT116 and SW620 cells with *SNORD18B/C* knockdown. Western blot analysis was performed on HCT116 and SW620 cells at 24 and 48 h post-*SNORD18B/C* siRNA transfection. **C**–**E** BRCA1 knockdown significantly inhibited HR repair. **C** Identification of HCT116 cells transfected with BRCA1, CtIP, or BLM and NC siRNA. **D** HR repair assay was performed in HCT116 cells transfected with BRCA1, CtIP, or BLM and NC siRNA (two-tailed Student’s *t* test, *n* = 3). **E** Immunofluorescence analysis of RAD51 in HCT116 cells with BRCA1 downregulation at 4 h post-10 Gy irradiation. Scale bar: 5 μm (Mann–Whitney test, *n* > 200 cells). **F**, **G** *SNORD18B/C* depends on BRCA1 for HR repair promotion. **F** Effect of BRCA1 knockdown on *SNORD18B/C* regulation of HR repair. HR reporter assays were performed in HCT116 cells transfected with NC and BRCA1 siRNAs across control, *SNORD18B* and *SNORD18C* overexpression groups (two-tailed Student’s *t* test, *n* = 3). **G** BRCA1 overexpression rescues HR deficiency induced by *SNORD18B/C* knockdown. An HR reporter assay was performed in HCT116 cells stably expressing LV-BRCA1 or LV-NC (negative control), and these cells were co-transfected with siRNA (siNC or si*SNORD18B/C*) and HR reporter plasmids. The number of GFP^+^ cells was measured 48 h post-transfection via flow cytometry. (two-tailed Student’s *t* test, *n* = 3). Mean ± SD, **P < 0.01, ***P < 0.001, *ns* means no significance.

HR repair, regulated by CDK1 kinase activity, primarily occurs in the S-phase and G2-phase cells due to CDK1’s inactivity in the early G1 phase [2]. We observed that *SNORD18B/C* overexpression did not elevate the proportion of cells in S phase or G2/M phase; instead, it caused a slight reduction (Fig. S6). The results indicate that *SNORD18B/C*’s effect on HR efficacy is independent of cell cycle regulation. To clarify the targets of *SNORD18B/C*, we performed a systematic analysis of core HR repair factors via western blotting. As shown by the results, 24 and 48 h post *SNORD18B/C* knockdown, critical early-stage HR factors, including BRCA1, CtIP, and BLM, were downregulated in both HCT116 and SW620 cells, accompanied by upregulation of γ-H2AX (a hallmark of DSB). However, the expression of primary NHEJ factors remained largely unaltered (Fig. 2A, B). Using DR-GFP reporter assays, we observed that the depletion of BRCA1, CtIP, or BLM compromised HR efficiency, among which BRCA1 knockdown showed the greatest impact on HR repair (Fig. 2C, D). Moreover, immunofluorescence analysis revealed that BRCA1 depletion markedly suppressed the formation of radiation-induced RAD51 foci (Fig. 2E). Given the critical role of BRCA1 in DSB end resection and the assembly of RAD51 nucleoprotein filaments, we speculated that BRCA1 could represent a primary target for *SNORD18B/C* in HR repair. To confirm this speculation, we assessed HR efficiency in *SNORD18B/C*-overexpressing cells with BRCA1 knockdown and revealed that BRCA1 depletion significantly attenuated *SNORD18B/C*-mediated regulation of HR repair. (the percentage of GFP^+^ cells in the siBRCA1 group: control 0.85 ± 0.06%, *SNORD18B* 0.97 ± 0.06%, *SNORD18C* 1.14 ± 0.04% and siNC group: control 2.02 ± 0.07%, *SNORD18B* 3.05 ± 0.10%, *SNORD18C* 3.10 ± 0.08%) (Fig. 2F). A reversal experiment revealed that restoring BRCA1 in *SNORD18B/C*-knockdown cells could rescue dysfunctional HR (Fig. 2G). In summary, these results confirmed that *SNORD18B/C* enhances HR repair primarily through regulating BRCA1 expression.

### *SNORD18B/C* regulates the translation of *BRCA1* mRNA in a non-2′-O-Me-dependent manner

**Fig. 3:**
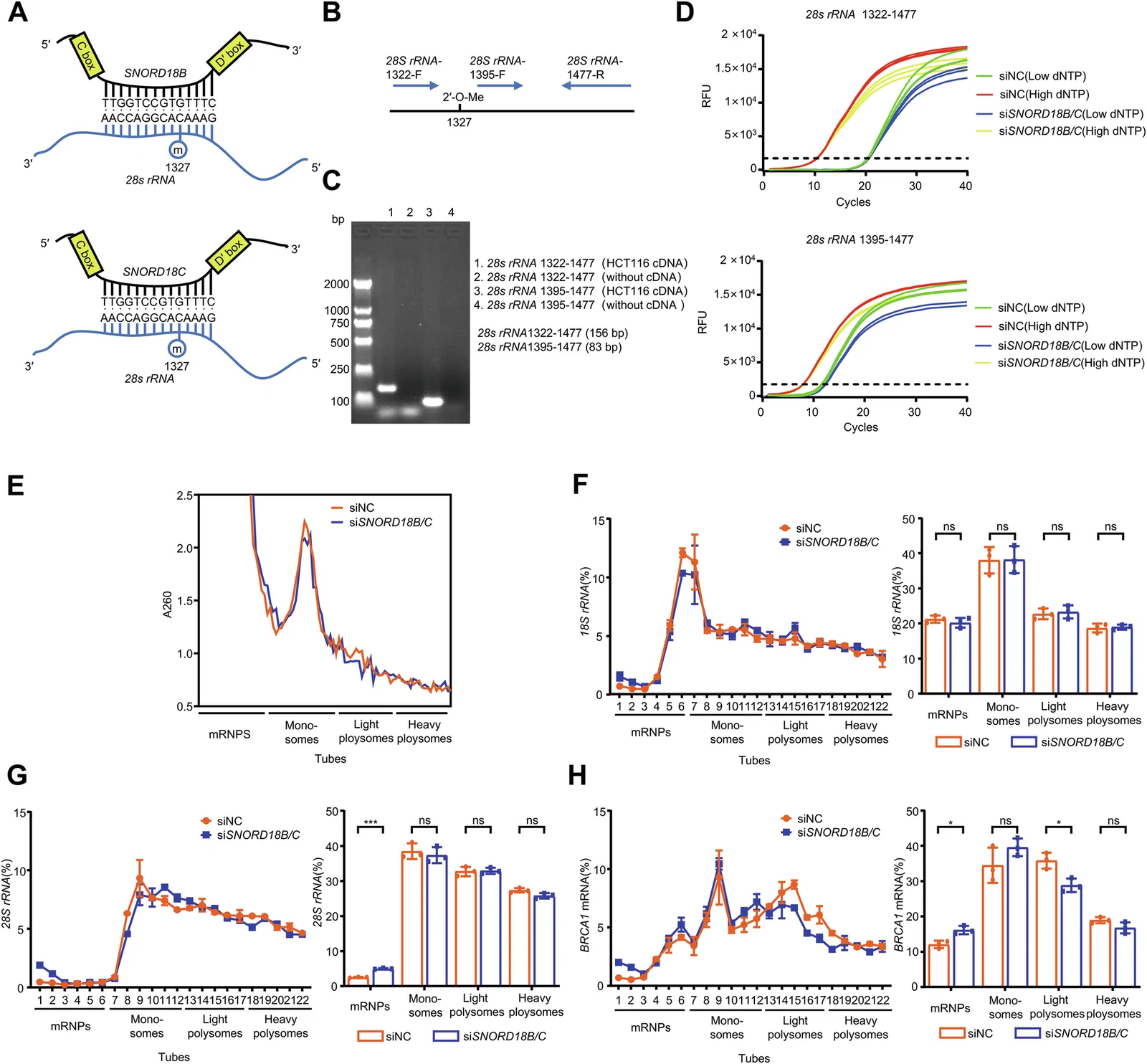
*SNORD18B/C* regulates the translation of *BRCA1* mRNA in a non-2′-O-Me-dependent manner. **A**–**D** *SNORD18B/C* knockdown does not alter 2′-O-Me at *28S rRNA* G1327 site. **A** The complementary pairing region between *SNORD18B/C* and *28S rRNA*, as well as the 2′-O-Me modification site of *28S RNA* at G1327. **B** Schematic of the primer design strategy for detecting the 2′-O-Me modification sites of *28S RNA* at G1327. The orientations of the primers are indicated by arrows. **C** Verification of DNA electrophoresis of PCR products. **D** Analysis of 2′-O-Me at *28S rRNA* G1327 by RTL-P assays. The *28S rRNA* G1327 site of 2′-O-Me under 1 mM (high) or 50 μM (low) dNTP conditions was detected by qPCR in *SNORD18B/C*-depleted HCT116 cells. **E**–**H** The impact of *SNORD18B/C* depletion on ribosome biogenesis and the translation of *BRCA1* mRNA. HCT116 cells were treated with cycloheximide, lysed, and fractionated on 10–50% sucrose gradients. **E** A260 nm fractionation profiles, **F** *18S rRNA*, **G** *28S rRNA*, and **H** *BRCA1* mRNA distribution in gradient fractions were detected by qRT-PCR (two-tailed Student’s *t* test, *n* = 3); *P < 0.05, ***P < 0.001, *ns* means no significance.

SnoRNAs regulate gene expression across multiple levels, including transcriptional, post-transcriptional, translational, and post-translational modification [15]. Quantitative analysis revealed no significant alteration in BRCA1 mRNA levels upon *SNORD18B/C* depletion (Fig. S7A). The BRCA1 protein is primarily degraded through the ubiquitin-mediated proteasomal pathway [34, 35]. In this study, we found that MG132, a proteasome inhibitor, failed to rescue the si*SNORD18B/C*-induced suppression of the BRCA1 protein. Consistently, interference with *SNORD18B/C* did not affect the BRCA1 protein attenuation in cycloheximide-treated cells (Fig. S7B, C). The canonical function of C/D box snoRNAs involves guiding 2′-O-Me of rRNA via their antisense elements positioned upstream of the D (or D′) box, thereby facilitating ribosome assembly and regulating mRNA translation [14, 15]. Does *SNORD18B/C* regulate *BRCA1* mRNA translation through this canonical pathway? Sequence alignment revealed that *SNORD18B/C* targets the G1327 of *28S rRNA* (Fig. 3A), a known methylation site [36, 37], implicating its involvement in 2′-O-Me. The Reverse Transcription at Low dNTP concentration followed by quantitative PCR (RTL-P) assay is an approach for detecting sites of 2′-O-Me in RNA molecules via reverse transcriptase stalling at modified nucleotides [38]. Specifically, low dNTP concentrations amplify methylation-induced reverse transcriptase stalling, whereas high dNTP concentrations overcome this stalling. To detect the 2′-O-Me at position 1327 of *28S rRNA*, we designed two forward primers (*28S rRNA*-1322-F and *28S rRNA*-1395-F) flanking the target methylation site, along with a common downstream reverse primer (*28S rRNA*-1477-R) (Fig. 3B). The target methylation site is located within the *28S rRNA* 1322–1477 region, but outside the 1395–1477 region. These primers successfully amplified the expected fragments (Fig. 3C). RTL-P assays confirmed methylation within the 1322–1395 region of *28S rRNA*. In the siNC group, the Δ*C*_q_ values (C_q low dNTPs_-C_q high dNTPs_) for the *28S rRNA* 1322–1477 amplicon (10.00 ± 0.09) were markedly higher than those for the *28S rRNA* 1395–1477 amplicon (3.82 ± 0.20). However, *SNORD18B/C* knockdown did not significantly alter the Δ*C*_q_ values for *28S rRNA* 1322–1477 amplicon (siNC 10.00 ± 0.09, si*SNORD18B/C* 10.21 ± 0.05) (Fig. 3D), indicating that *SNORD18B/C* does not affect 2′-O-Me at position 1327 of *28S rRNA*. Sucrose gradient density centrifugation analysis of ribosome biosynthesis revealed that *SNORD18B/C* knockdown did not impair ribosome assembly, and the binding capabilities of *18S rRNA* and *28S rRNA* with polysomes remained largely unaffected, indicating minimal impact on global protein translation (Fig. 3E-G). Interestingly, in *SNORD18B/C*-knockdown cells, the enrichment of *BRCA1* mRNA in polysomes was significantly reduced (Light polysomes: siNC 35.42 ± 2.26%, si*SNORD18B/C* 28.44 ± 1.95%) (Fig. 3H). These findings indicate that *SNORD18B/C* depletion disrupts polyribosome association with *BRCA1* mRNA, thereby suppressing BRCA1 translation through a mechanism independent of rRNA 2′-O-Me regulation.

### *SNORD18B/C* directly interacts with ribosomal protein RPL23A to promote HR repair by regulating BRCA1 expression

**Fig. 4:**
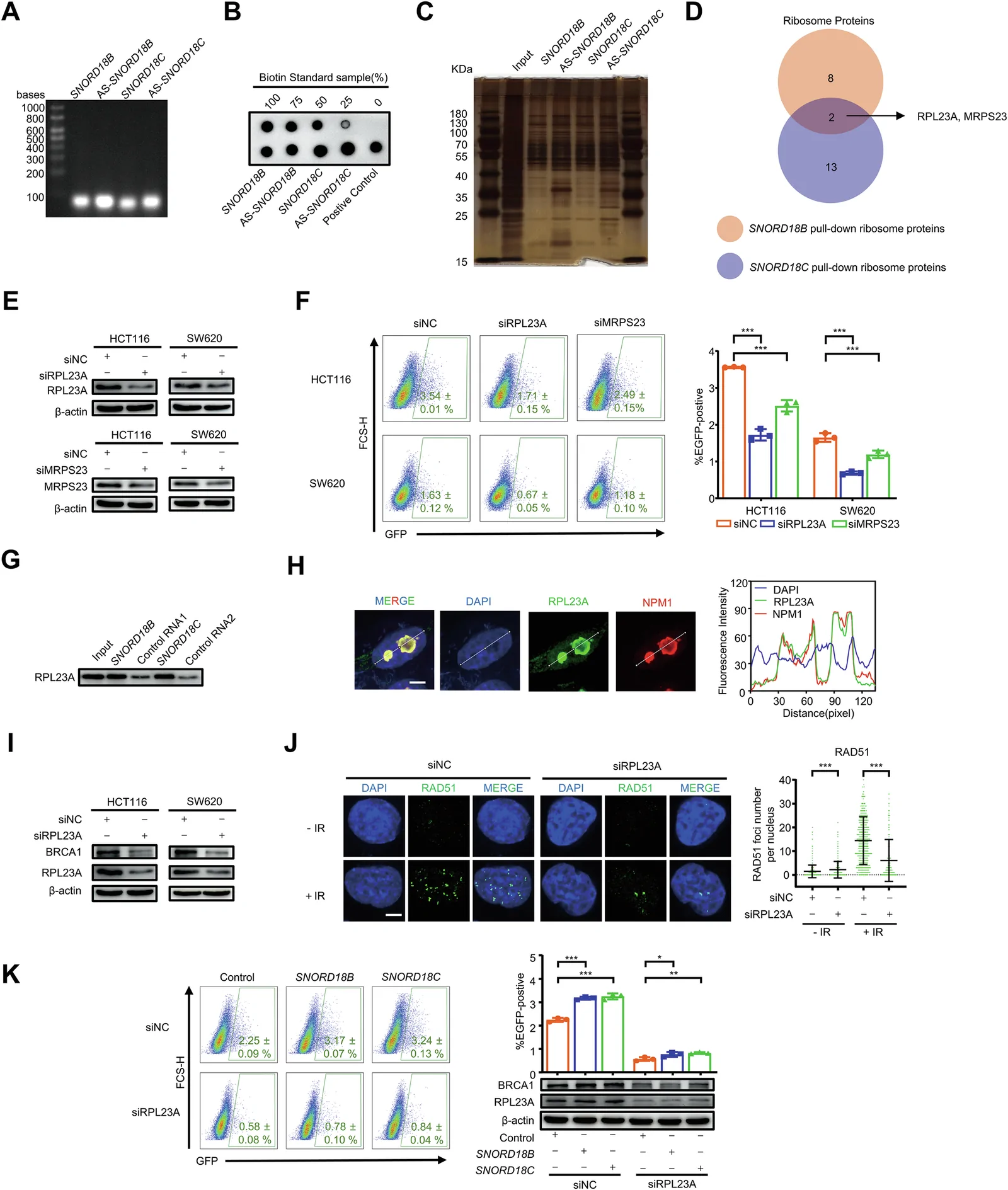
*SNORD18B/C* directly interacts with ribosomal protein RPL23A to promote HR repair by regulating BRCA1 expression. **A**–**D** Biotin-labeled *SNORD18B/C* RNA pulldown. **A** RNA electrophoresis. *SNORD18B/C* and its antisense RNAs were generated by in vitro transcription using T7 promoter-containing DNA templates. The transcribed RNAs were purified and analyzed by electrophoresis. **B** The detection of biotinylated RNA. The transcribed RNA was processed through 5′ end desulfurization biotin labeling, and labeling efficacy was assessed. **C** Silver-stain electrophoresis analysis of the RNA pulldown lysate. The biotin-labeled RNAs were utilized to pull down target proteins from the cell lysates, followed by gel electrophoresis. **D** A Venn diagram comprises ribosomal proteins interacting with *SNORD18B* (upper, MS Signals^*SNORD18B*^ ≥ 1.5-fold of MS Signals^*AS-SNORD18B*^) or *SNORD18C* (lower, MS Signals^*SNORD18C*^ ≥ 1.5-fold of MS Signals^*AS-SNORD18C*^). RPL23A and MRPS23 were located in the intersection (middle). **E**, **F** Knocking down RPL23A significantly inhibited HR repair in CRC cells. **E** Identification of HCT116 and SW620 cells with MRPS23 and RPL23A knockdown. Western blotting was performed on HCT116 and SW620 cells at 48 h post-siRNA transfection. **F** HR repair analysis was assessed in MRPS23- or RPL23A-depleted HCT116 and SW620 cells (mean ± SD, two-tailed Student’s *t* test, *n* = 3). **G** Identification of the *SNORD18B/C*–RPL23A interaction via an RNA pulldown assay. Immunoblot analysis of RPL23A in the *SNORD18B/C* RNA pulldown lysate. **H** RPL23A is predominantly localized to the nucleolus. HCT116 cells were transfected with the RPL23A-Myc recombinant plasmid. Myc-tag (Green) and NPM1(Red) were co-labeled via immunofluorescence assay, followed by line-scan fluorescence intensity quantification using ImageJ software. **I** RPL23A knockdown reduced BRCA1 expression. Western blotting was performed on HCT116 and SW620 cells at 48 h post-siRNA transfection. **J** Immunofluorescence analysis of radiation-induced RAD51 foci formation. HCT116 cells with RPL23A downregulation were exposed to 10 Gy γ-ray irradiation (62.3 cGy min^−1^) and collected 4 h post-irradiation. Scale bar: 5 μm (Mann–Whitney test, *n* > 200 cells). **K** Silencing RPL23A attenuated *SNORD18B/C*-mediated HR regulation. An HR reporter assay was performed on HCT116 cells stably infected with LV-NC, LV-*SNORD18B*, or *SNORD18C*. The cells were co-transfected with siRNAs (siNC or siRPL23A) and HR reporter plasmids, followed by flow cytometry analysis of HR efficiency at 48 h post-transfection (mean ± SD, two-tailed Student’s *t* test, *n* = 3). *P < 0.05, **P < 0.01 ***P < 0.001.

RNA–protein interaction represents a fundamental mechanism underlying snoRNA regulation of DNA damage repair. Does *SNORD18B/C* directly interact with specific proteins to modulate BRCA1 translation? To validate our hypothesis, we performed an RNA pull-down assay using in vitro-transcribed *SNORD18B/C* sense transcripts, with antisense transcripts as negative controls, to enrich for physically interacting proteins (Fig. 4A). After biotinylation and validation (Fig. 4B), the transcripts were immobilized on streptavidin beads and used to pull down *SNORD18B/C*-interacting proteins from CRC lysates, visualized by silver staining after sodium dodecyl sulfate–polyacrylamide gel electrophoresis (Fig. 4C). Protein samples were analyzed by 4D label-free quantitative mass spectrometry, and differentially bound proteins were identified using the following cutoff criteria: MS signals for *SNORD18B* or *SNORD18C* ≥ 1.5-fold of those for the antisense control (AS-*SNORD18B/C*). Based on previous evidence, we focused on ribosomal proteins interacting with *SNORD18B/C*. Mass spectrometry revealed that *SNORD18B* and *SNORD18C* bound to 10 and 15 ribosomal proteins, respectively, sharing only two common interactors: RPL23A and MRPS23 (Fig. 4D and Table S1). HR assays showed that RPL23A depletion more significantly impaired HR efficiency in both HCT116 and SW620 cells compared to MRPS23 depletion, as evidenced by a greater reduction in GFP^+^ cell populations (Fig. 4E, F). Consequently, this led us to focus specifically on the role of RPL23A in the *SNORD18B/C*-mediated HR process in the present study. First, a direct interaction between *SNORD18B/C* and RPL23A was confirmed by RNA pull-down assay (Fig. 4G). Furthermore, immunofluorescence assays revealed spatial colocalization of RPL23A with the nucleolar marker NPM1 (Fig. 4H), providing subcellular evidence for the RPL23A–*SNORD18B/C* interaction, given *SNORD18B/C*’s nucleolar localization. Given the crucial role of RPL23A in the regulation of translation, we analyzed its impact on BRCA1 expression. The data revealed that RPL23A depletion significantly reduced BRCA1 expression and, as expected, suppressed ionizing radiation-triggered RAD51 foci formation, indicating RPL23A’s involvement in the BRCA1-mediated HR repair (Fig. 4I, J). An even more pressing question that remains to be explored is whether RPL23A modulates the effect of *SNORD18B/C* on HR efficiency. In further investigations, we observed that RPL23A depletion attenuated *SNORD18B/C*-mediated HR enhancement, accompanied by reduced BRCA1 (Fig. 4K). In summary, these findings indicate that the ribosomal protein RPL23A is critically involved in regulating BRCA1 expression. Moreover, RPL23A directly interacts with *SNORD18B/C* and modulates its biological effect on BRCA1-mediated HR repair.

### *SNORD18B/C* regulates the translation of BRCA1 via RPL23A–*BRCA1* mRNA interactions

**Fig. 5:**
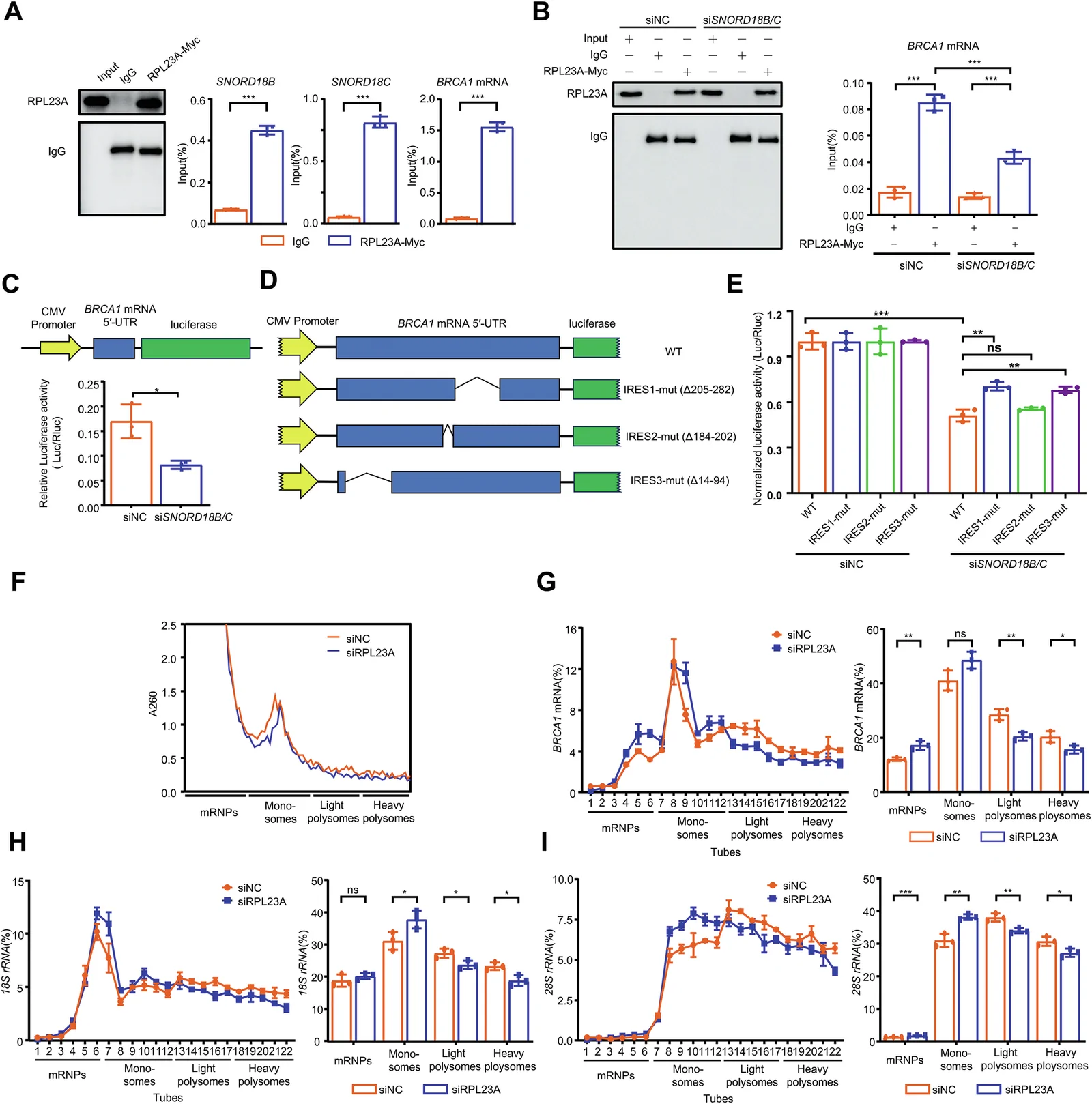
*SNORD18B/C* regulates the translation of BRCA1 via RPL23A–BRCA1 mRNA interactions. **A** RPL23A coimmunoprecipitated with both *SNORD18B/C* and BRCA1 mRNAs in HCT116 cells. RIP analysis was conducted in the cells overexpressing RPL23A-Myc tag protein. Western blot images of RIP products with antibodies against the Myc tag and IgG as a negative control (left). RIP-qPCR assays of *SNORD18B*, *SNORD18C*, and BRCA1 in RPL23A immunoprecipitates (right). **B** *SNORD18B/C* depletion reduced RPL23A binding affinity to *BRCA1* mRNA. RIP-qPCR assessed RPL23A binding to BRCA1 mRNA in *SNORD18B/C* knockdown and control cell lysates. **C** *SNORD18B/C* knockdown suppresses *BRCA1* mRNA 5′UTR translation efficiency. A luciferase reporter plasmid containing the *BRCA1* mRNA 5′UTR was constructed. Cells were co-transfected with *SNORD18B/C* siRNA and the reporter plasmid (Luciferase/Renilla luciferase), followed by quantitative analysis of translational activity. **D** Schematic representation of the fluorescent reporter plasmids harboring mutations in the IRES of the *BRCA1* mRNA 5′UTR. The wild-type (WT) plasmid contains *BRCA1* mRNA 5′UTR, whereas the IRES1-mut, IRES2-mut, and IRES3-mut plasmids harbor mutations in *BRCA1* mRNA 5′UTR regions 205–282, 184–202, and 14–94, respectively. **E** Translation efficiency of the *BRCA1* mRNA 5′UTR IRES mutants upon *SNORD18B/C* knockdown. **F**–**I** *SNORD18B/C* knockdown suppresses *BRCA1* mRNA translation. HCT116 cells were treated with cycloheximide, lysed, and fractionated on 10–50% sucrose gradients. **F** A260 nm fractionation profiles, **G** *BRCA1* mRNA, **H** *18S rRNA*, and **I** *28S rRNA* distribution in gradient fractions analyzed by qRT-PCR. (two-tailed Student’s *t* test, *n* = 3). *P < 0.05; **P < 0.01, ***P < 0.001, *ns* means no significance.

To determine whether the *SNORD18B/C*-RPL23A complex regulates the translation of BRCA1 protein through a direct interaction with its mRNA, A RIP assay with HCT116 cells expressing RPL23A-Myc was performed. RPL23A exhibited significantly higher binding affinity for both *SNORD18B/C* and *BRCA1* mRNAs compared to the IgG control (*SNORD18B*: 0.447 ± 0.021% versus IgG: 0.067 ± 0.002%, *SNORD18C*: 0.814 ± 0.044% versus IgG: 0.062 ± 0.004%, *BRCA1* mRNA: 1.576 ± 0.071% versus IgG: 0.109 ± 0.013%) (Fig. 5A). These findings indicated that RPL23A can simultaneously bind to the *SNORD18B/C* and *BRCA1* mRNAs, forming a stable RNA-protein ternary complex. Our investigations on the mutual regulation between *SNORD18B/C* and RPL23A demonstrated that their expression is independently controlled (Fig. S8A, B). This finding suggested that the *SNORD18B/C*-RPL23A-*BRCA1* mRNA complex serves as the structural basis for BRCA1 translation and HR repair. To further elucidate the role of *SNORD18B/C* in mediating RPL23A–*BRCA1* mRNA interaction, we measured the affinity of RPL23A for *BRCA1* mRNA upon *SNORD18B/C* depletion. The results revealed that *SNORD18B/C* knockdown significantly reduced the affinity between RPL23A and *BRCA1* mRNA (Fig. 5B). Our results suggest that *SNORD18B/C* dysregulation disrupts the stable binding between RPL23A and *BRCA1* mRNA, leading to the suppression of BRCA1 translation and HR repair.

The internal ribosome entry site (IRES) within mRNA 5′ untranslated region (5′UTR) serves as a translation element for ribosome–mRNA interactions, regulating the translation of specific mRNAs required for DNA damage repair [39–41]. According to the prediction from the Human IRES Atlas website based on four distinct transcript variants of the *BRCA1* gene, the 5′UTR of *BRCA1* mRNA contains four IRES sites (Table S2). Moreover, bioinformatic analysis using the RIPSeq database revealed a strong binding affinity between RPL23A and *BRCA1* mRNA 5′UTR. Specifically, SVM (Support Vector Machine) and RF (Random Forest) classifiers show binding scores of 0.94 and 0.85, respectively, both exceeding the 0.5 binding potential threshold (Table S3). These findings suggest that RPL23A exhibits strong binding affinity to the *BRCA1* mRNA 5′UTR, implicating RPL23A in IRES-dependent translational regulation of BRCA1. This study constructed a *BRCA1* mRNA 5′UTR-incorporated luciferase plasmid to assess translational activity, with reporter assays confirming *SNORD18B/C* knockdown significantly inhibited *BRCA1* mRNA 5′UTR translational activity (siNC:0.170 ± 0.034, si*SNORD18B/C* 0.082 ± 0.008) (Fig. 5C). Using luciferase reporter assays, we validated the roles of three predicted IRES elements (IRES1–3) within the *BRCA1* transcript sequence (NM_001407571.1) employed in this study, with respect to *SNORD18B/C*-mediated regulation of BRCA1 translation (Fig. 5D). The results revealed that mutations in IRES1 and IRES3 attenuated the *SNORD18B/C*-mediated regulation of the *BRCA1* 5′UTR (Fig. 5E), suggesting that this regulation is partially dependent on IRES1 and IRES3. Finally, polysome profiling confirmed that RPL23A knockdown significantly reduced the polysomal loading of *BRCA1* mRNA (Light polysomes: siRPL23A 19.99 ± 1.45% versus siNC 27.99 ± 2.01%; Heavy polysomes: siRPL23A 15.21 ± 1.43% versus siNC 19.89 ± 2.12%), attenuating its translation while exerting a moderate inhibitory effect on global protein synthesis (Fig. 5F–I). Collectively, our research demonstrated that *SNORD18B/C* enhances RPL23A–*BRCA1* mRNA interaction by directly binding to RPL23A, thereby modulating BRCA1 translation to improve HR repair.

### *SNORD18B/C* enhances proliferation and radioresistance in CRC cells

**Fig. 6:**
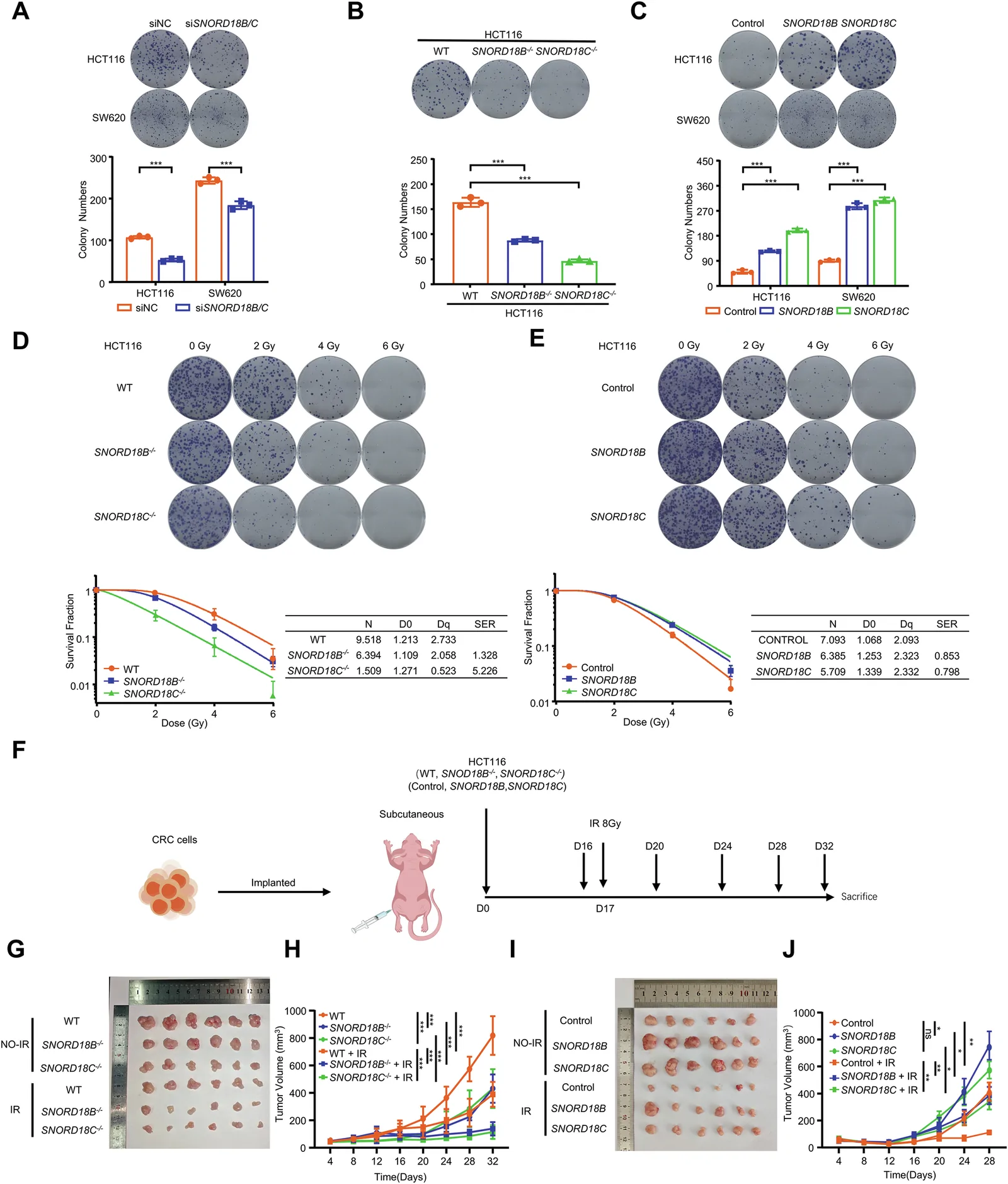
*SNORD18B/C* enhances proliferation and radioresistance in CRC cells. **A**–**C** *SNORD18B/C* promoted colony formation in CRC cells. Colony formation assays in HCT116 or SW620 cells with *SNORD18B/C* knockdown (**A**), knockout (**B**), and upregulation (**C**). HCT116 and SW620 cells were cultured in a six-well plate at a density of 800 cells per well for 10–12 days. The cells were fixed with methanol, stained with Giemsa dye, and counted (two-tailed Student’s *t* test, *n* = 3). **D**, **E** *SNORD18B/C* regulation of radiosensitivity. Radiosensitivity to a gradient dose of radiation (0, 2, 4, or 6 Gy) was measured in HCT116 cells with (**D**) *SNORD18B/C* knockout or (**E**) overexpression. Radiosensitivity was analyzed using the N, D0, Dq, and SER values from a multi-target single-hit model. **F**–**J** *SNORD18B/C* enhances CRC xenograft radioresistance in vivo. **F** Schematic representation of radiation exposure in nude mice with transplanted tumors. CRC cells with *SNORD18B/C* knockout or overexpression were subcutaneously injected into nude mice. When tumor volume reached ~100 mm^3^ (~16 days post-injection), mice were subjected to local tumor irradiation (8 Gy) or sham irradiation. Tumor volumes were measured every 4 days. After ~1 month, the mice were euthanized, and the xenografts were harvested and weighed. **G**, **I** Representative images of CRC tumors harvested from nude mice. **H**, **J** Longitudinal monitoring of tumor volume was performed at 4-day intervals (two-tailed Student’s *t* test, *n* = 6). *P < 0.05, **P < 0.01, ***P < 0.001, *ns* means no significance.

DSB caused by DNA replication stress strictly depends on the HR pathway, hence making HR essential for maintaining cell proliferation and survival. Using CCK-8 assays, we observed that both *SNORD18B/C* knockdown and knockout significantly suppressed cell proliferation in HCT116 and SW620 cells, while its overexpression enhanced growth capacity (Figs. S9A–C and S2D). Colony formation assays revealed that *SNORD18B/C* knockdown significantly reduced colony numbers in CRC cells (HCT116: si*SNORD18B/C* 50.67 ± 3.21, siNC 104.33 ± 3.21; SW620: si*SNORD18B/C* 180.67 ± 9.50, siNC 239.67 ± 7.51) (Fig. 6A). Similarly, an inhibitory effect was also observed in *SNORD18B/C* knockout cells (HCT116^*SNORD18B*−/−^ 87.67 ± 2.52, HCT116^*SNORD18C*−/−^ 46.33 ± 3.21, HCT116^wild-type^ 164.33 ± 9.07) (Fig. 6B). Conversely, *SNORD18B/C* overexpression significantly increased colony formation (HCT116: *SNORD18B* 120.67 ± 3.06, *SNORD18C* 195.67 ± 7.02, control 47.33 ± 6.81; SW620: *SNORD18B* 282.67 ± 10.79, *SNORD18C* 304.33 ± 9.87, control 87.00 ± 4.36) (Fig. 6C).

To determine whether *SNORD18B/C*’s regulation of the HR pathway protects cells from radiation-induced DSB damage, we investigated its impact on cell survival post-ionizing radiation using a colony formation assay. Compared to wild-type HCT116 cells, the surviving fraction of cells with *SNORD18B* or *SNORD18C* knockout was significantly reduced following exposure to gradient doses of γ-rays, indicating increased cell radiosensitivity, with sensitizer enhancement ratio (SER) values exceeding 1 (SER of HCT116^*SNORD18B*−/−^ = 1.328, SER of HCT116^*SNORD18C*−/−^ = 5.226) (Fig. 6D). In contrast, *SNORD18B/C* overexpression enhanced cellular radioresistance, as evidenced by SER values of 0.853 and 0.798 for *SNORD18B* and *SNORD18C*, respectively (Fig. 6E). We analyzed the effects of *SNORD18B/C* on the apoptosis induced by γ-ray irradiation and hydrogen peroxide (a genotoxic oxidizing agent that induces DNA damage). The results revealed that *SNORD18B/C* overexpression markedly suppressed apoptosis induced by DNA damage (Fig. S10). To further explore the roles of *SNORD18B/C* in tumor radiosensitivity in vivo, we established xenograft models using CRC cells with *SNORD18B/C* knockout or overexpression, followed by irradiation. Xenografts with *SNORD18B* or *SNORD18C* knockout exhibited significantly higher radiosensitivity than control xenografts (Figs. 6F–H and S11A–C). Conversely, *SNORD18B* or *SNORD18C* overexpression significantly enhanced tumor radioresistance (Figs. 6I, J and S11E–G). Ki-67 IHC staining also revealed that *SNORD18B/C* promoted tumor cell proliferation upon irradiation (Fig. S11D, H). In conclusion, *SNORD18B/C* enhances cellular resistance to both endogenous and exogenous DSB-induced stress via HR repair, thereby promoting oncogenesis by modulating proliferation and radioresistance.

### High expression of *SNORD18B/C* correlates with poor prognosis in CRC patients

**Fig. 7:**
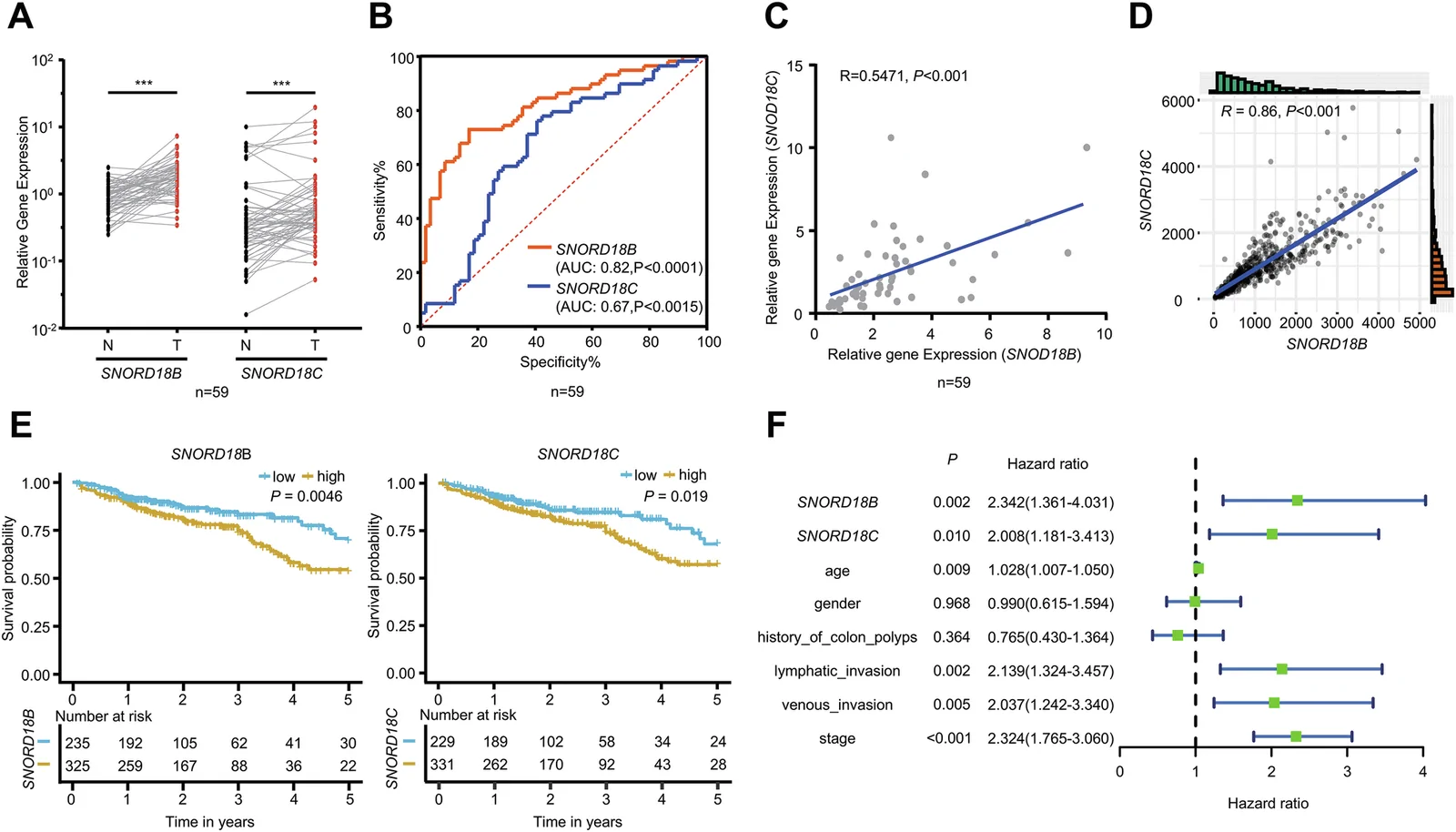
High expression of *SNORD18B/C* correlates with poor prognosis in CRC patients. **A** *SNORD18B/C* expression in paired CRC and adjacent normal tissues was determined via qRT-PCR (Wilcoxon test, *n* = 59, ***P < 0.001). **B** ROC curve analysis of *SNORD18B/C* expression in paired CRC and adjacent normal tissues (Wilcoxon test, *n* = 59). **C** Correlation analysis of the expression of *SNORD18B* and *SNORD18C* in CRC tissues (Pearson’s correlation analysis, *n* = 59). **D** Correlation analysis of *SNORD18B* and *SNORD18C* expression in CRC tissues from the TCGA database (Pearson’s correlation analysis, *n* = 593). **E** High expression of *SNORD18B* and *SNORD18C* was associated with poor overall survival. Kaplan–Meier curve of the 5-year overall survival analysis in the *SNORD18B/C* high- and low-expression groups from the TCGA database (log-rank test, *n* = 560). **F** Cox proportional hazards regression model via univariate analysis was performed to measure the risk of survival (*n* = 410). ***P < 0.001.

To investigate *SNORD18B/C*’s clinical significance in CRC, we analyzed its expression in clinical CRC tissues and found significantly elevated levels in cancerous tissues compared to adjacent normal tissues (Fig. 7A). ROC analysis results indicated that *SNORD18B/C* levels effectively differentiated CRC tissues from adjacent normal tissues (Fig. 7B). In addition, Pearson correlation analysis revealed a significant positive correlation between the expression levels of the two genes (Fig. 7C, D). Using the optimal cutoff level of *SNORD18B/C* expression, we stratified CRC cohorts from the TCGA database into high and low-expression groups. Kaplan–Meier analysis demonstrated significantly reduced overall survival in CRC patients exhibiting elevated *SNORD18B/C* expression compared to their low-expression counterparts (Fig. 7E). Moreover, Cox regression analysis identified high *SNORD18B/C* expression as an independent risk factor for poor prognosis (Fig. 7F). These data suggest that *SNORD18B/C* could serve as a potential target against CRC.

## Discussion

BRCA1-mediated HR repair is crucial for maintaining genomic stability by repairing DSB or protecting stalled DNA replication forks [42]. SnoRNAs were originally classified as functionally conserved small noncoding RNAs (ncRNAs) involved in the regulation of post-transcriptional modifications. While accumulating evidence demonstrates their participation in diverse biological processes, their specific contribution to HR repair remains poorly characterized [15, 43, 44]. This is the first study to investigate the function of the radiation-responsive snoRNA genes *SNORD18B* and *SNORD18C*, which directly interact with the ribosomal protein RPL23A, an uncharacterized factor in DNA damage repair, and regulate the BRCA1-mediated HR repair via the *SNORD18B/C*–RPL23A–BRCA1 axis.

Extensive evidence substantiates that snoRNAs contribute to oncogenesis by modulating tumor cell proliferation, invasion, apoptosis, and metastasis [45]. However, the regulatory mechanisms of snoRNAs in radiosensitivity remain inadequately investigated. In 2022, Rastorgueva et al. [46] profiled snoRNA expression dynamics post-X-ray irradiation in two distinct leukemia cell models with differential radiosensitivity, but lacked functional validation of these differentially expressed snoRNAs. In 2023, Chen et al. [47] first discovered that altered *SCARNA2* expression affects cell sensitivity to ^60^Co γ-ray radiation. Additionally, recent findings from Liu et al. [28] revealed that *SNORA28* modulates CRC cell radiosensitivity through the LIFR/JAK1/STAT3 pathway. In the present study, we identified two homologous radiation-responsive snoRNA genes, namely *SNORD18B* and *SNORD18C*, providing the first functional characterization of these two molecules. *SNORD18B/C* enhances proliferation and radioresistance in CRC cells, identifying it as a novel oncogenic factor. The DNA repair capacity serves as a fundamental determinant of tumor radiosensitivity. While ncRNAs’ roles in the process of DNA repair have been extensively studied, the involvement of snoRNAs has only been discovered in recent years. Bergstrand et al. [17] demonstrated that *SCARNA2* suppresses the NHEJ pathway by regulating the phosphorylation-mediated activation of DNA-PKcs, thus indirectly increasing HR efficiency to promote DSB repair. Intriguingly, our study identifies *SNORD18B* and *SNORD18C* as novel regulators that directly enhance HR repair, providing new insights into snoRNA-mediated DNA damage repair in response to irradiation.

As a critical regulator of HR repair, BRCA1 is involved in the generation of free 3′ ssDNA ends through direct interaction with CtIP and BARD1, subsequently recruiting recombinase RAD51 to mediate DNA strand invasion [1, 9, 10]. In our study, the data reveal that *SNORD18B/C* depletion downregulates BRCA1 expression, consequently attenuating DSB-induced RAD51 foci formation, indicating that *SNORD18B/C* contributes to BRCA1-mediated 3′ ssDNA end processing and RAD51 recruitment. Based on current research, this constitutes the inaugural evidence that snoRNAs regulate HR repair via direct modulation of BRCA1 expression. BRCA1-associated HR deficiency is conventionally attributed to *BRCA1* gene mutations; however, emerging evidence identifies other types of ncRNAs involved in the epigenetic regulation of BRCA1. For instance, lncRNAs *CISAL* and *MALAT1* bind with transcriptional regulatory factors GABPA and EZH2, respectively, inhibiting *BRCA1* gene transcription [48, 49]. *MiR-182* and *MiR-9* silence *BRCA1* mRNA by targeting the 3′UTR region [50, 51], while *circPLEKHM3* acts as a competing endogenous RNA to antagonize *miR9*-mediated *BRCA1* mRNA repression [52]. Notably, our results establish a novel function for *SNORD18B/C* in regulating BRCA1 protein synthesis through polyribosome loading, a mechanism independent of *28S rRNA* 2′-O-Me. While this is not the first description of snoRNA-mediated translational regulation [18, 53], this finding holds considerable importance for revealing a new layer of regulation in the HR repair pathway.

Furthermore, the present study provides the first evidence that ribosomal protein RPL23A is a crucial regulator of HR repair. Ribosomal proteins are primarily recognized for their canonical role in ribosome assembly and protein biosynthesis. Moreover, a subset of them participates in DNA damage repair through distinct mechanisms: modulating translation or interacting with specific proteins in a ribosome-independent manner. As reported by Chuanzhen et al. [22] and Yun-long et al. [23], ribosomal protein L6 and ribosomal protein P0 directly bind to H2A and NONO proteins, respectively, thereby modulating the DSB repair processes. Conversely, RPL26 specifically regulates p53 translation through a direct interaction with the 5′UTR of its mRNA following DNA damage induced by ultraviolet irradiation [20]. Our research found that RPL23A directly binds with *BRCA1* mRNA to regulate BRCA1 translation, thereby enhancing HR repair through a canonical ribosome-dependent way. Meanwhile, we found that *SNORD18B/C* directly binds to RPL23A and facilitates the interaction of RPL23A with *BRCA1* mRNA. Building upon Cheng et al.’s study of snoRNA–ribosomal protein interactions in cellular senescence regulation [24], our study provides strong evidence that this interplay also critically impacts DNA damage repair.

Then, how does *SNORD18B/C*-RPL23A mediate the regulation of BRCA1 translation? Existing evidence indicates that DNA damage induces selective translation of mRNAs through IRES-mediated ribosome interactions, maintaining essential protein synthesis under stress conditions [21, 54]. Notably, our study indicates that the 5′UTR of *BRCA1* mRNA contains two IRES sites (nt 205–282 and 14–94), both of which are required for the *SNORD18B/C*-RPL23A complex in the selective translation of *BRCA1* mRNA during radiation-induced DSB repair. Concurrently, we observed that mutations at these two sites did not completely abolish the inhibitory effect of *SNORD18B/C* knockdown on BRCA1 translational activity, implying that additional unidentified regulatory sites may exist. Due to the poor specificity of *SNORD18B/C* probes for fluorescence in situ hybridization, there is no evidence available to support the role of an *SNORD18B/C*-RPL23A-*BRCA1* mRNA ternary complex in regulating the selective translation of BRCA1. The precise underlying mechanisms, particularly the ternary complex’s tri-molecular target sites and HR repair-associated core domains, require further investigation.

This study identifies radiation-responsive snoRNA *SNORD18B/C* as a novel promoter of HR repair through regulation of BRCA1 translation, leading to enhanced cell proliferation, radioresistance, and poor prognosis in CRC. We firstly confirmed that *SNORD18B/C* directly interacts with RPL23A to modulate BRCA1 translation, thereby revealing a novel snoRNA-ribosomal protein-mediated regulatory mechanism for the HR repair pathway. These findings provide new potential therapeutic targets for CRC treatment.

## Materials and methods

### Clinical specimens

Clinical specimens of cancer tissues and adjacent normal tissues from patients with pathologically confirmed CRC were collected from Liaoning Cancer Hospital (Shenyang, China) and stored at −80 °C. All samples and clinical information were obtained with the approval of the Ethics Committee on Human Investigation of the Liaoning Cancer Hospital (20190970), with written informed consent provided by all participants. A cohort comprising 59 matched pairs of tissue samples was analyzed using quantitative reverse transcription polymerase chain reaction (qRT-PCR) assays. All primers are listed in Table S4. Detailed information regarding all the samples is provided in Table S5.

### Cell culture

CRC cell lines (HCT116 and SW620) were purchased from GeneChem (Shanghai, China). HCT116 and SW620 cells were maintained in RPMI-1640 medium (Sigma, R8758, Missouri, USA) supplemented with 10% fetal bovine serum (ExCell Bio, FSP500, Shanghai, China) and 1% Penicillin–Streptomycin Solution (HyClone, SV30010, Utah, USA). All the cells were cultured in a humidified incubator at 37 °C with 5% CO_2_. For ionizing radiation treatment, CRC cells were exposed to γ-rays with doses ranging from 2 to 10 Gy at a dose rate of 62.3 cGy min^−1^ by ^60^Co γ-ray irradiation equipment. For protein stability analysis, HCT116 cells were treated with DMSO or 10 μM MG132 for 12 h; HCT116 cells were exposed to 100 μg mL^−1^ cycloheximide or DMSO for 0, 2, 4, 8, 12, or 24 h.

### SiRNA and plasmid transfection

All the gene-specific siRNA duplexes used in this study are listed in Table S6. SiRNA oligonucleotides were transfected at a final concentration of 80 nM (GenePharma, Suzhou, China) using jetPEI reagent (Polyplus, 101000046, Strasbourg, France) as specified. The plasmids were transiently transfected into cells with jetPEI reagent following the manufacturer’s instructions. The following analyses were performed 24 h and 48 h post-transfection.

### Lentiviral transfection

Lentiviruses (GV235) expressing *SNORD18B* (LV-*SNORD18B*), *SNORD18C* (LV-*SNORD18C*), or negative control oligonucleotide sequences (LV-NC) were constructed by GeneChem. The RNA sequence information is listed as follows: LV-NC (5′–3′): UUCUCCGAACGUGUCACGU; LV-*SNORD18B* (5′–3′): UCAAAAUGAUGAGAUUCCACUUAAUUGGUCCGUGUUUCUGAAACACAUGAUAUUUGUGGAAAUUCUGACU; LV-*SNORD18C* (5′–3′): UUGUUAUGAUGAGAUUCCACUUAAGGUCCGUGUUUCUGAAACAAAUGAUUUUGUGGAAGUUCUGAUU.

The plasmids used for expression of the empty vector or BRCA1 (pLVX-BRCA1; MiaoLing Bio, P27743 and P32447, Wuhan, China) were packaged into lentiviruses by GeneChem Corporation. The cells were cultured in 12-well plates (6 × 10^4^ cells/well) for 24 h before being infected with lentiviruses at a multiplicity of infection of 10. Then, the cells were cultured in selection medium containing puromycin (Solarbio; P8230, Beijing, China, 2 μg mL^−1^) for 72 h after infection. The stably infected cell lines were then maintained in medium supplemented with puromycin (0.5 μg mL^−1^) for further research.

### RNA isolation and qRT-PCR

Total RNA was isolated from cells and tissues using TRIzol reagent (Sigma, T9424). Reverse transcription of complementary DNA (cDNA) was conducted with a PrimeScript Reverse Transcriptase Reagent Kit (TaKaRa, RR047Q, Shiga-ken, Japan). cDNA samples were amplified and quantified in triplicate by SYBR Green PCR Master Mix (Mei5Bio, MF787, Beijing, China) with a real-time PCR detection system (Bio-Rad, CFX96, California, USA). *ACTB* and *U6* served as endogenous controls. The primers used in this study are listed in Table S4.

### CRISPR/Cas9 gene editing

The *SNORD18B/C* gene was knocked out in HCT116 cells using CRISPR/Cas9 gene-editing technology. A pair of specific short guide RNA sequences targeting the 5′- and 3′-UTRs of each gene was selected from the ChopChop website (https://chopchop.cbu.uib.no/). These sequences were cloned and inserted into the pX459 vector (Addgene, 62988, Massachusetts, USA), and the plasmids were transfected into HCT116 cells. Twenty-four hours after transfection, the cells were passaged and cultured in selection medium containing puromycin (2 μg mL^−1^) for 24 h. The selected cells were counted and seeded at a density of one cell per well in 96-well plates. Four weeks later, the expression of *SNORD18B/C* was analyzed by qRT-PCR, and *SNORD18B* or *SNORD18C* knockout cell lines (HCT116^*SNORD18B*−/−^ or HCT116^*SNORD18C*−/−^) were acquired and stored in liquid nitrogen.

### Cell proliferation assay and colony formation assay

For the cell proliferation assay, 1000 cells/well were plated in 96-well plates in quintuplicate. Cell proliferative activity was measured using a CCK-8 kit (Dojindo, CK04, Kumamoto Ken, Japan) according to the manufacturer’s instructions. The optical density was measured at 450 nm (OD_450_) with a microplate reader (Tecan, Sunrise, Switzerland). For the colony formation assay, cells were seeded in triplicate in six-well plates (500–1000 cells/well). After 10–12 days of incubation, the colonies were fixed with anhydrous methanol for 30 min, stained with Giemsa for 1 h, and then counted by Fiji software.

### Flow cytometric assay

For apoptosis analysis, the cells were collected and washed twice with PBS. Annexin V-FITC apoptosis detection kit (Dojindo, AD10) was used to assess apoptosis. For each sample and in triplicate for each group, 10,000 cells were analyzed by flow cytometry (ACEA NovoCyte, 2060 R, California, USA). In cell cycle analysis, the cells were fixed with 70% (v/v) ethanol at −20 °C for 24 h. The fixed cells were washed twice with PBS, followed by incubation in propidium iodide solution containing ribonuclease. In total, 20,000 cells were analyzed by flow cytometry for each sample and in triplicate for each group.

### Western blotting

The cells were lysed in lysis buffer (0.1% SDS, 0.5% sodium deoxycholate, and 1% NP-40 in PBS) supplemented with complete protease inhibitor cocktail tablets (Roche, 04693132001, Basel, Switzerland) and phosphatase inhibitor cocktail tablets (Roche, 04906837001). The protein concentration of the extract was quantified using a BCA protein assay kit (Beyotime, P0012AC, Beijing, China). For western blotting, 20–40 μg protein samples were subjected to SDS-PAGE and then transferred to nitrocellulose membranes (PALL, T42800, New York, USA). The membranes were incubated overnight at 4 °C with specific primary antibodies, followed by 2 h of incubation with peroxidase-conjugated secondary antibodies at room temperature. The antigen-antibody reactions were visualized by West Pico PLUS Chemiluminescent Substrate (Thermo Fisher, 34580, Massachusetts, USA) in an Image Quant LAS AI800 system (GE Healthcare, Illinois, USA). The antibodies used are listed in Table S7.

### Dual-luciferase reporter assay

The *BRCA1* mRNA 5′UTR (NM_001407571.1) and its IRES mutants were inserted into the pGL3 reporter vector to generate the recombinant plasmids pGL3-BRCA1 5′UTR and the corresponding IRES mutants. The plasmids were constructed by Tsingke Biotechnology. The sequences of *BRCA1* mRNA 5′UTR and its IRES mutants are listed in Table S8. Cells were seeded into 12-well plates (1.2 × 10^4^/well) for 24 h and transfected with NC or *SNORD18B/C* siRNA. After 8 h, cells were co-transfected with 1.25 μg the recombinant plasmid of pGL3-BRCA1 5′UTR and its IRES mutants and 0.125 μg pRL-CMV internal control plasmid. Following 48 h incubation, cell lysates were analyzed using a Dual-Luciferase Reporter Assay System (Promega, E1910, Wisconsin, USA). Firefly luciferase activity was normalized to Renilla luciferase activity with triplicate measurements per group.

### NHEJ and HR assays

The NHEJ and HR assay protocols were described previously [55]. For the NHEJ assay, cells were co-transfected with 1.25 μg of linearized EJ5-GFP plasmid (digested with the endonuclease HindIII) and 0.25 μg of pCherry plasmid in 12-well plates. After 24 h of transfection, the cells were collected, and the percentage of GFP-positive (GFP^+^) cells was quantified using a flow cytometer. For the HR assay, cells were co-transfected with 0.75 μg DR-GFP plasmids and 0.75 μg I-SceI plasmids in 12-well plates. The cells were harvested 48 h after transfection, and the proportion of GFP^+^ cells was measured by flow cytometry. For lentivirus-infected cells, NHEJ and HR reporter assays were performed as described above. For the siRNA-transfected cells, the cells were transfected with a specific siRNA twice, both before and after the transfection of the NHEJ and HR reporter gene plasmids.

### Immunofluorescence

HCT116 and SW620 cells subjected to different treatments were cultivated on sterilized coverslips in six-well plates for 72 h. The cells were fixed in 4% formaldehyde for 15 min at 2 or 4 h post 10 Gy irradiation, permeabilized with PBS buffer containing 0.3% Triton X-100, and then blocked with 5% bovine serum albumin for 1 h at room temperature. The cells were subsequently incubated overnight with RAD51 antibody (Abcam; ab150116, 1:500, Cambridge, UK) at 4 °C in a humidified chamber and washed three times with PBST. Next, the coverslips were incubated with a goat anti-rabbit IgG antibody (Alexa Fluor 488) (Abcam; ab150077, 1:200) at room temperature for 2 h and washed three times. The nuclei were stained with antifade mounting medium containing 4′,6-diamidino-2-phenylindole (ZSGB-BIO, ZLI-9556, Beijing, China). Images were recorded with a Nikon TI2-E X-LIGHT V3 laser confocal microscope imaging system. The number of foci in each nucleus was quantified using Fiji software.

### RNA pulldown

*SNORD18B*, *SNORD18C*, and the antisense sequences were amplified to DNA containing the T7 promoter by PCR. The DNA was transcribed to RNAs in vitro using the Large Scale RNA Production System (Promega, P1300) according to the manufacturer’s guidelines. Subsequently, the RNAs were purified by phenol/chloroform reagent. The quality of these RNA extracts was assessed through electrophoresis on a denaturing agarose gel. The RNA samples were biotinylated by the RNA 3′ End Desthiobiotinylation Kit (Thermo Fisher, 20163) and detected by the Scientific Chemiluminescent Nucleic Acid Detection Module Kit (Thermo Fisher, 17097). Biotinylated RNAs were employed to capture specific proteins from cell lysates using the Magnetic RNA-Protein Pull-Down Kit (Thermo Fisher, 20164). The protein samples obtained from RNA pulldown were subjected to mass spectrometric analysis by Genechem, and the results are shown in Table S1.

### RNA immunoprecipitation

The pcDNA3.1-RPL23A-Myc plasmid was transiently transfected into HCT116 cells. Forty-eight hours later, the transfected cells (1 × 10^7^) were harvested and lysed with 500 μL of lysis buffer [20 mM Tris-HCl pH 8.0, 137 mM NaCl, 1% NP40, 2 mM EDTA, protease inhibitor cocktail (Selleck, B14001, Texas, USA), and RNase inhibitor (Beyotime, R0102)]. Following centrifugation, the cell supernatants were precleared with 5% fetal bovine serum and Protein A/G PLUS-Agarose (Santa Cruz, sc-2003, Texas, USA) for 1 h. Subsequently, 10% of the sample was reserved as an input control, and the remainder was incubated with an anti-Myc-tag antibody (Proteintech, 16286, Wuhan, China) or IgG (Beyotime, A7016) overnight at 4 °C. The RNA-antibody complexes were incubated with Protein A/G PLUS-Agarose at 4 °C for 4 h, after which the RNA-antibody-bound agarose beads were collected and washed with washing buffer [10 mM Tris-HCl (pH 7.4), 1 mM EDTA, 1 mM EGTA (pH 8.0), 150 mM NaCl, 1% Triton X-100, 0.2 mM sodium orthovanadate, and an RNase inhibitor (200 U mL^−1^)] three times. Finally, 20% of the sample was used for western blotting, and the remaining portion was used for RNA extraction and qRT-PCR analysis.

### RTL-P assay

The RTL-P assay was employed to detect 2′-O-Me at nucleotide G1327 of 28S rRNA as described in a previous publication [38]. The reverse transcription mixture consisted of 100 ng of total RNA, 50 μM specific RT primers, M-MLV RT reaction buffer, 200 U reverse transcriptase (Promega, M1701), 40 U RNase inhibitor, and a low (50 μM) or high (1 mM) concentration of deoxyribonucleoside triphosphates. The mixture was incubated for 1 h at 42 °C, and the reaction was terminated at 70 °C for 15 min. cDNA amplification was quantified by qRT-PCR.

### Polysome analysis

HCT116 cells were seeded at 1.2 × 10^6^ cells/well in 100-mm dishes and transfected with siRNA oligos (si*NC*, si*BRCA1*, or si*RPL23A*). After 48 h of transfection, the cells were incubated with 100 μg mL^−1^ cycloheximide for 10 min and then collected in ice-cold PBS supplemented with 100 μg mL^−1^ cycloheximide. The cells were lysed with gradient buffer (150 mM KCl, 20 mM HEPES, pH 7.6, and 6.5 mM MgCl_2_) supplemented with 1 mM DTT, 100 μg mL^−1^ cycloheximide, 0.5% Nonidet P-40, protease, and RNase inhibitors. The cell supernatants were harvested by centrifugation (12,000×*g* for 10 min at 4 °C). The supernatants were loaded onto a linear 10–50% sucrose gradient and centrifuged (28,000 rpm for 1.5 h at 4 °C) by ultracentrifugation (Hitachi, CP100NX, Tokyo, China). Eighty-eight fractions were collected from the top to the bottom of the gradient. The ribosome distribution was analyzed by measuring the absorbance of each fraction at an optical density of 260 nm using a UV spectrophotometer (Thermo Fisher, NanoDrop One). After combining every four fractions in order, RNA was isolated with TRIzol and then subjected to a qRT-PCR assay for further analysis.

### Xenograft tumor assay in nude mice

For the xenograft experiments, 5-week-old male BALB/c nude mice were purchased from Beijing SPF Biotechnology (Beijing, China) and randomly assigned to different cell line groups (*n* = 6 per group). Xenograft tumors were established using CRC cell lines with *SNORD18B/C* overexpression or knockout, as well as their corresponding control cells. Each mouse was subcutaneously injected with ~1 × 10^7^ cells suspended in 100 μL of PBS. When the tumor volume reached ~100 mm^3^, the mice in each cell line group were further randomized into irradiated and non-irradiated subgroups. Mice in the irradiation subgroups received local tumor irradiation with a single dose of 8 Gy. Tumor growth was monitored by measuring tumor volumes every 4 days using digital calipers. Tumor volume was calculated using the following formula: volume = (length) × (width)^2^ × (π/6). After ~1 month, the mice were euthanized, and the xenografts were harvested and weighed. The excised xenograft tumors were subjected to RT-qPCR analysis, hematoxylin and eosin (H&E) staining, and Ki67 immunohistochemistry (IHC). H&E staining and Ki67 IHC were performed by Solarbio (Beijing, China). SlideViewer and ImageJ software were used to view the digital slides and quantify the percentage of Ki67-positive cells. All animal procedures were conducted in accordance with the regulations of the Ethics Committee (IACUC-DWZX-2020-549) of the Academy of Military Medical Science (AMMS, China).

### Bioinformatic database

Data on CRC patients were obtained from The Cancer Genome Atlas (TCGA) website (https://portal.gdc.cancer.gov/). The expression profiles of *SNORD18B/C* and data concerning the 5-year overall survival of patients were sourced from the SNORic database (http://bioinfo.life.hust.edu.cn/SNORic).

### Statistical analysis

Data are presented as the mean ± standard deviation (SD). All experiments were conducted in triplicate or as three independent experiments. Two-tailed Student’s *t* tests were applied to qRT-PCR (*n* = 3), CCK-8 (*n* = 5), colony formation, and flow cytometry data. RAD51 foci counts (immunofluorescence) were analyzed using the Mann–Whitney test. *SNORD18B/C* expression in paired CRC and adjacent normal tissues was assessed by the Wilcoxon test; ROC curves and Pearson’s correlation analysis were additionally applied to these data. TCGA cohorts were evaluated using Pearson’s correlation analysis, survival analysis (Kaplan–Meier plotter, log-rank test), and the Cox proportional hazards regression. Significance threshold: *P* < 0.05; *ns* means no significance.

## Supplementary information

Supplementary Information

Supplemental Table

Original Data of western blots

## Data Availability

The mass spectrometry data of *SNORD18B/C* RNA pulldown are available via ProteomeXchange with identifier PXD059571. All data supporting the findings of this study are available in the main text and supplementary materials. The original full-size Western blots are shown in the Original Data of Western Blots. All cell lines, nucleotide sequences, or plasmids used in the study are available from the corresponding authors upon reasonable request.
