# Supplementary material for: SNORD18B/SNORD18C-RPL23A promotes homologous recombination repair by regulating BRCA1 translation in colorectal cancer cells
Source: Cell Death Discov. 2026 Aug 6;12:436. doi: 10.1038/s41420-026-03289-3 (PMC13731662; doi:10.1038/s41420-026-03289-3)
Supplement: Supplementary file 1 [file 41420_2026_3289_MOESM3_ESM.pdf]

Figure 2B

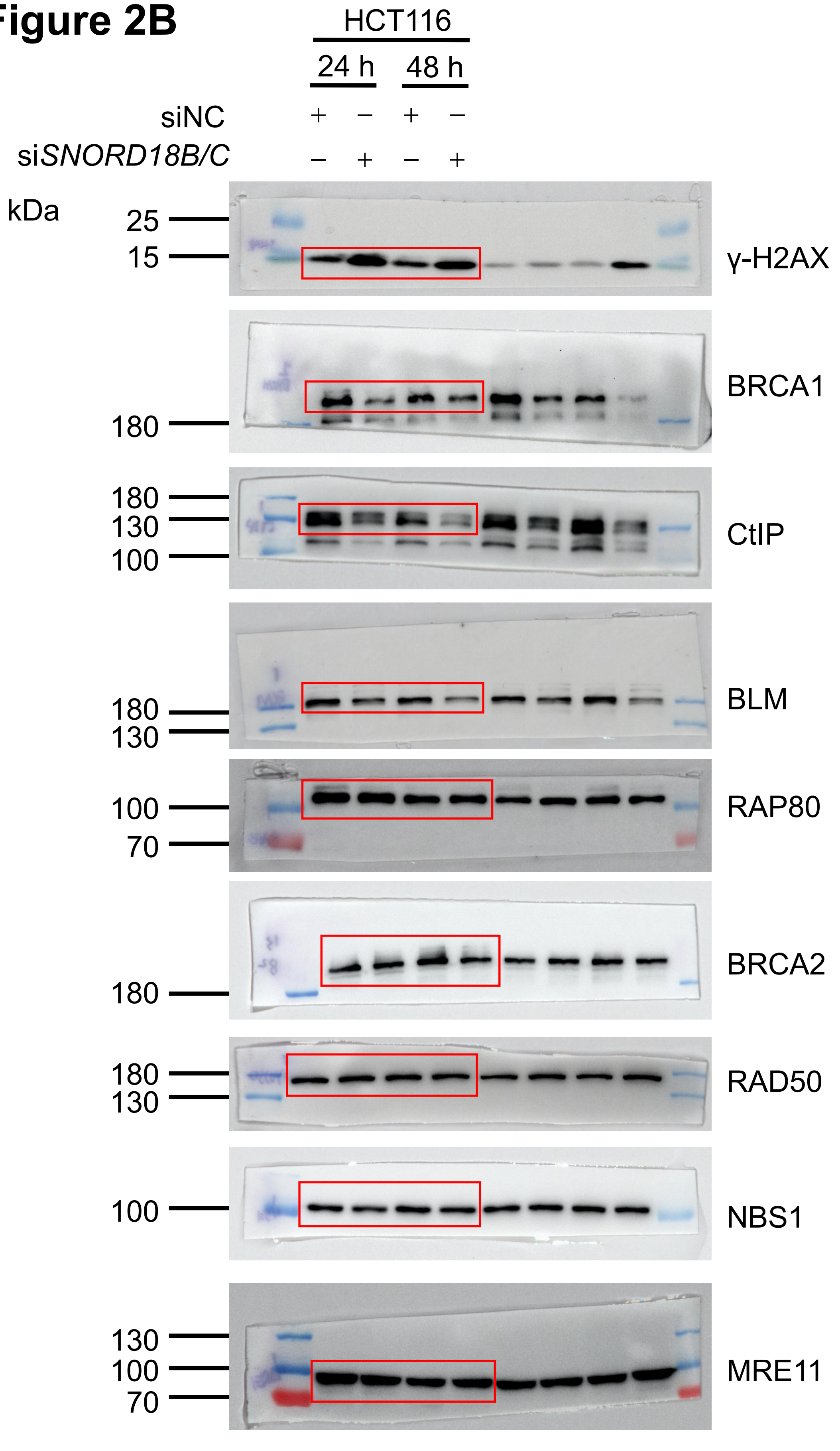

HCT116
24 h
48 h
siNC + − + −
siSNORD18B/C − + − +
kDa
25
15
γ-H2AX
180
BRCA1
180
130
100
CtIP
180
130
BLM
100
70
RAP80
180
BRCA2
180
130
RAD50
100
NBS1
130
100
70
MRE11

Figure 2B

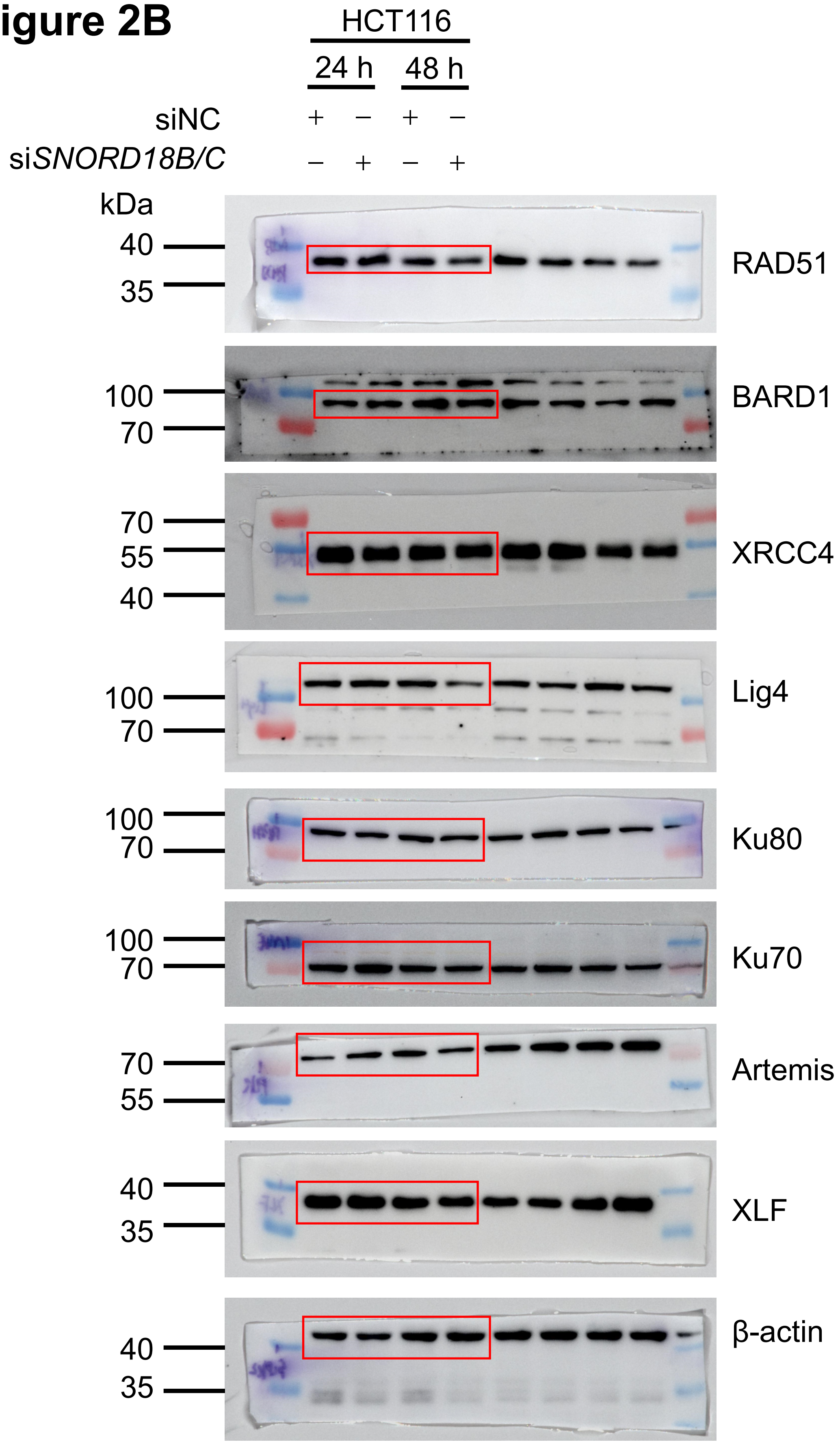
HCT116
24 h
48 h
siNC + − + −
si*SNORD18B/C* − + − +
kDa
40
35
RAD51
100
70
BARD1
70
55
40
XRCC4
100
70
Lig4
100
70
Ku80
100
70
Ku70
70
55
Artemis
40
35
XLF
40
35
β-actin

Figure 2B

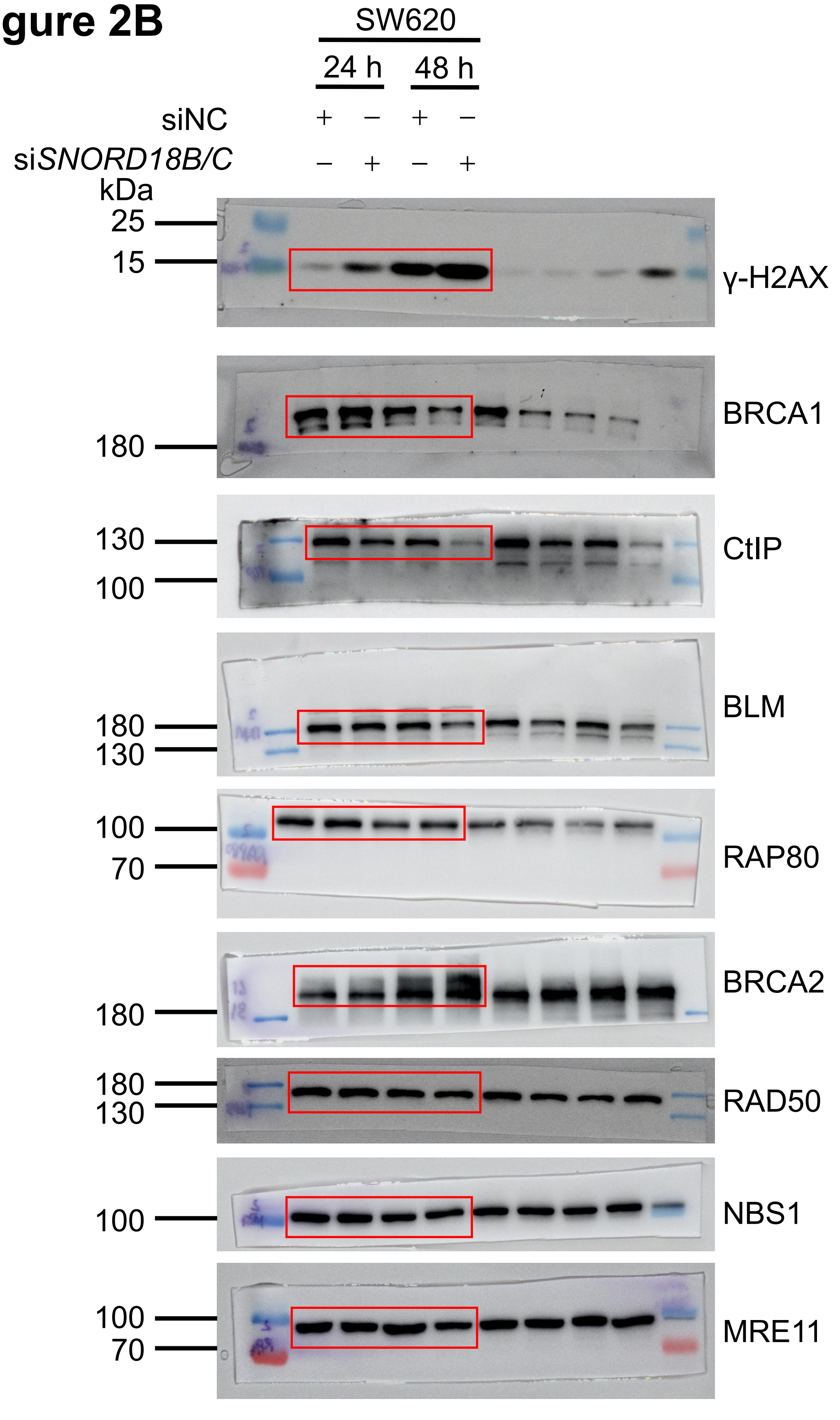
SW620
24 h
48 h
siNC
+ − + −
si*SNORD18B/C*
− + − +
kDa
25
15
γ-H2AX
BRCA1
180
130
100
CtIP
BLM
180
130
100
70
RAP80
BRCA2
180
180
130
RAD50
100
NBS1
100
70
MRE11

Figure 2B

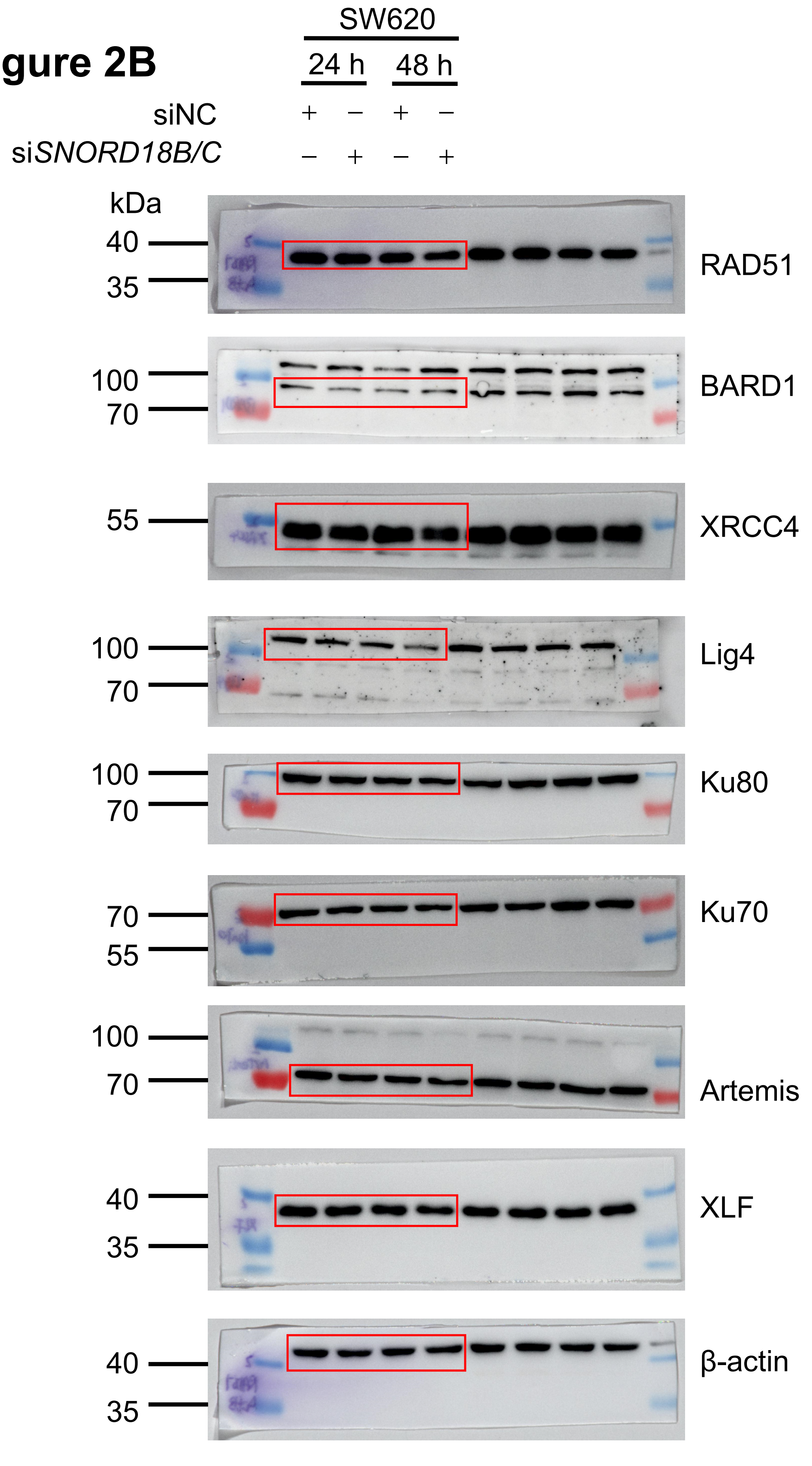
SW620
24 h
48 h
siNC + − + −
si*SNORD18B/C* − + − +
kDa
40
35
RAD51
100
70
BARD1
55
XRCC4
100
70
Lig4
100
70
Ku80
70
55
Ku70
100
70
Artemis
40
35
XLF
40
35
β-actin

Figure 2C

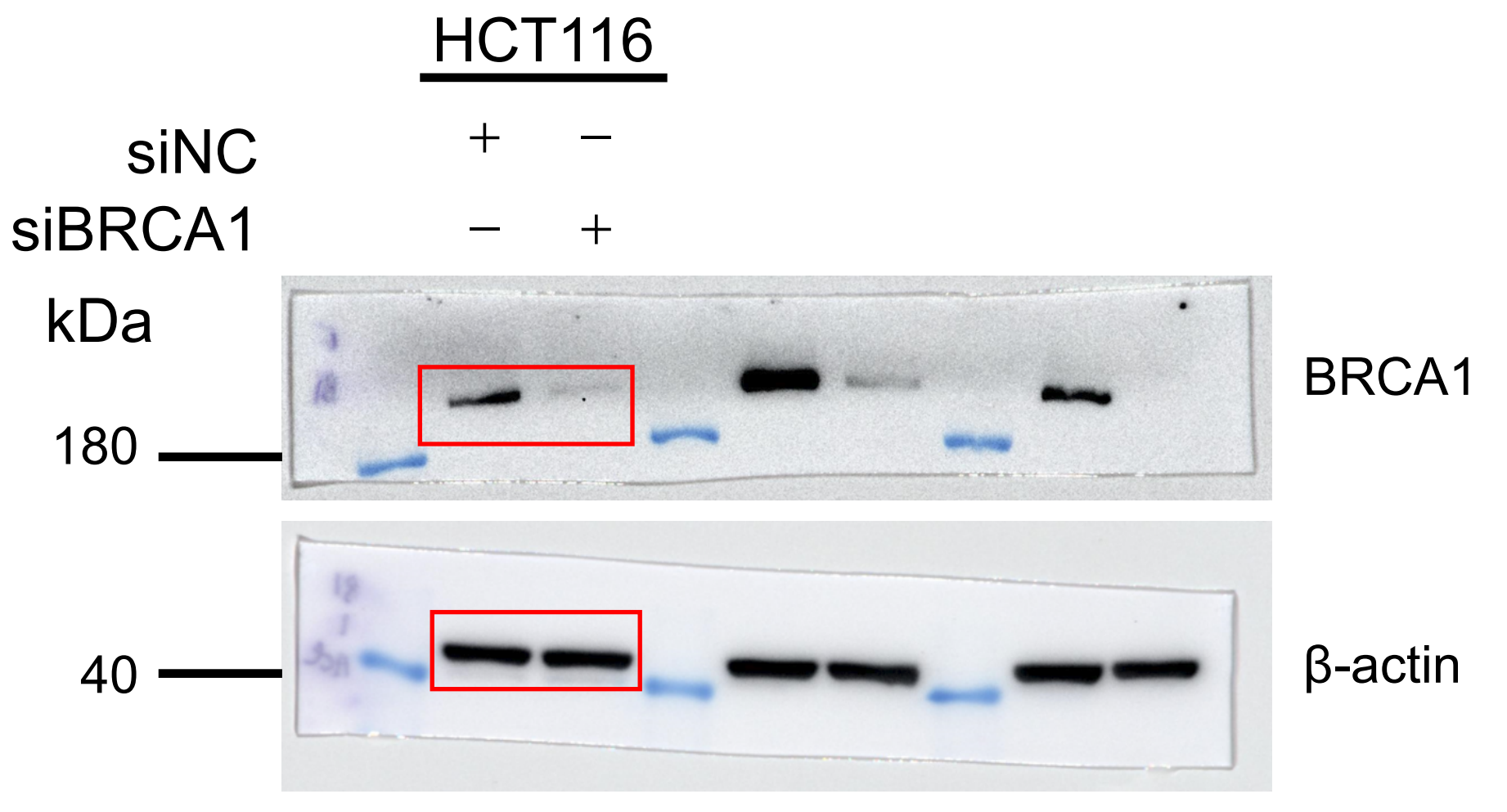
HCT116
siNC + −
siBRCA1 − +
kDa
180
40
BRCA1
β-actin

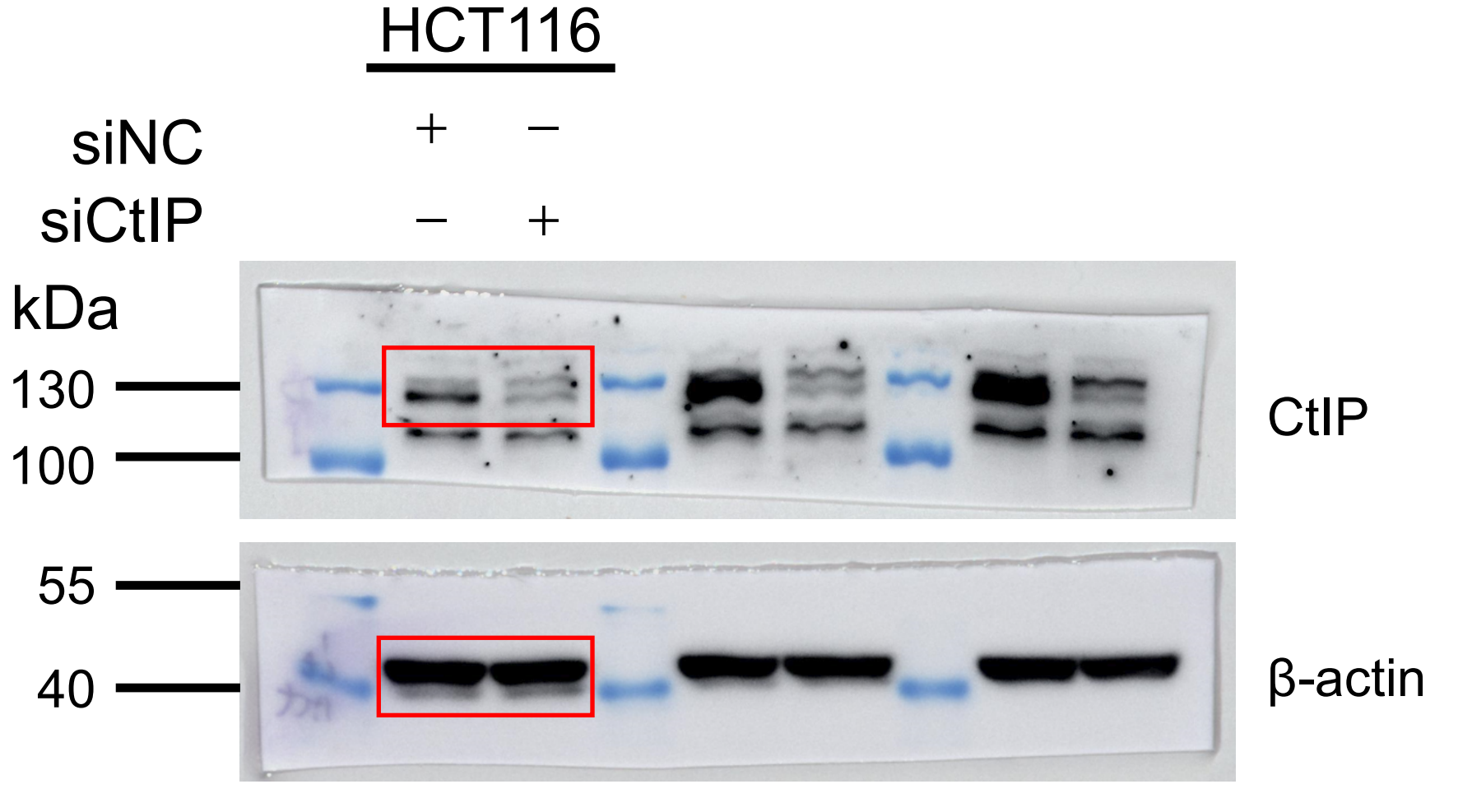
HCT116
siNC + −
siCtIP − +
kDa
130
100
55
40
CtIP
β-actin

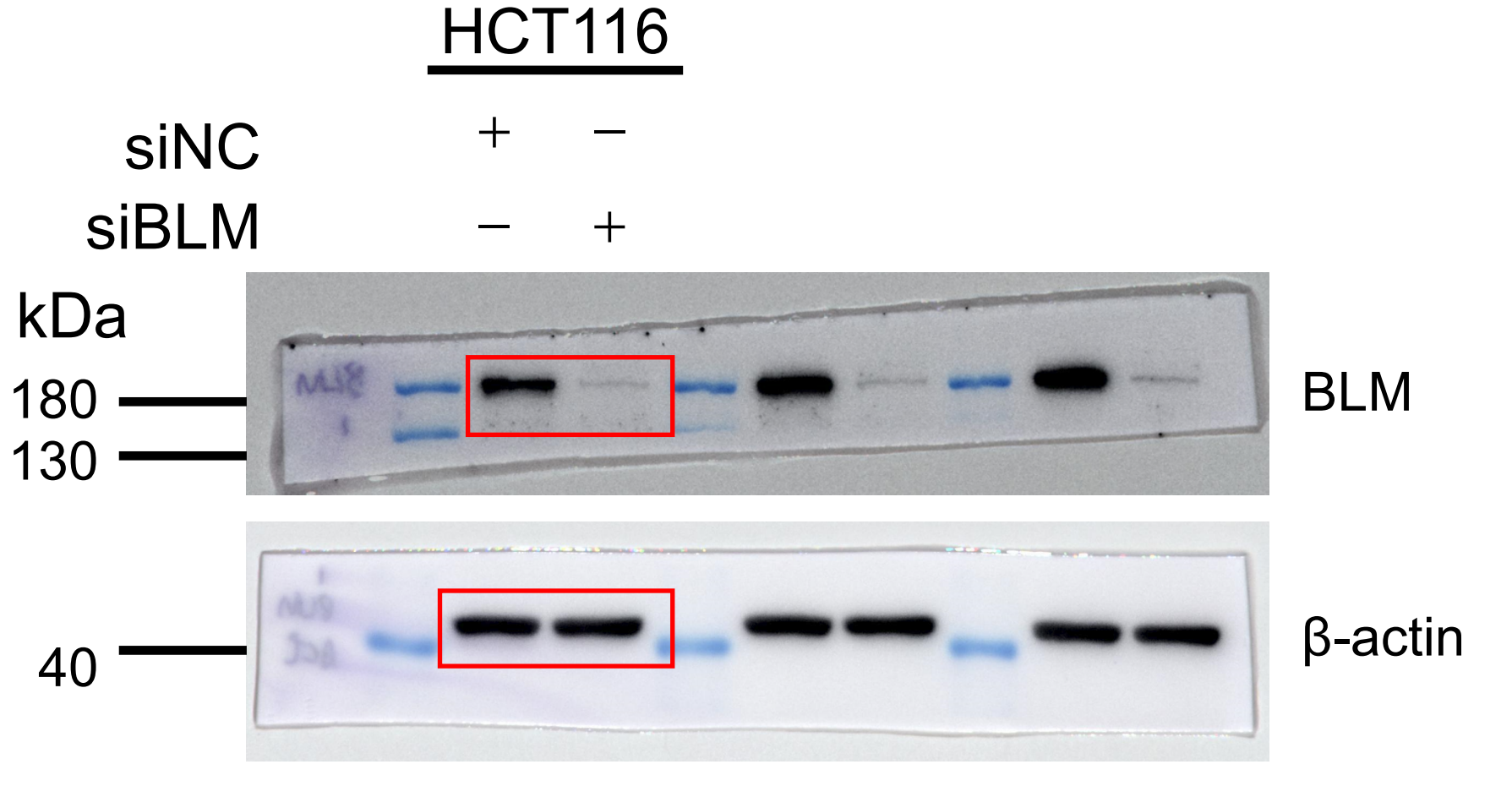
HCT116
siNC + −
siBLM − +
kDa
180
130
40
BLM
β-actin

Figure 2F

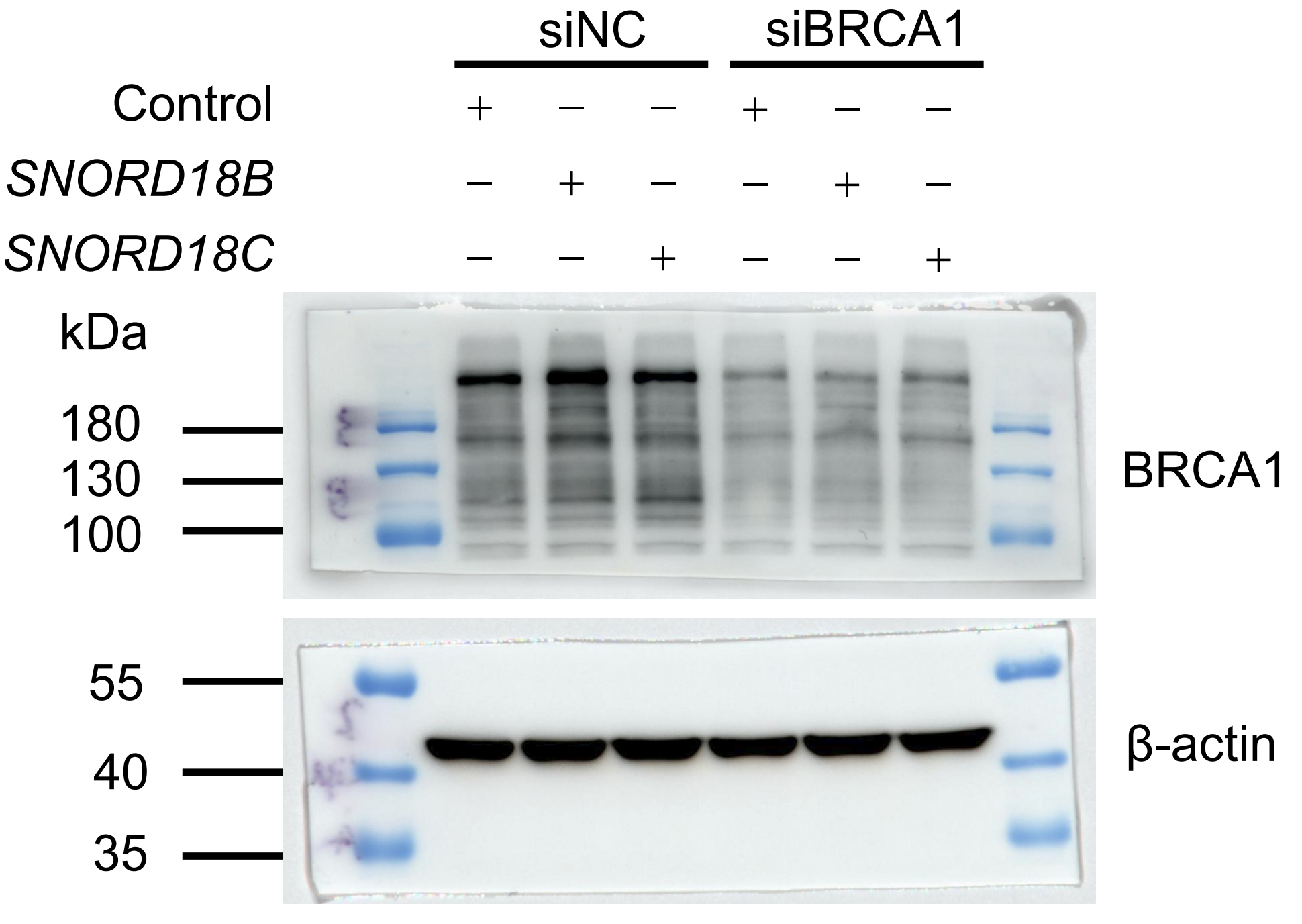

Figure 2G

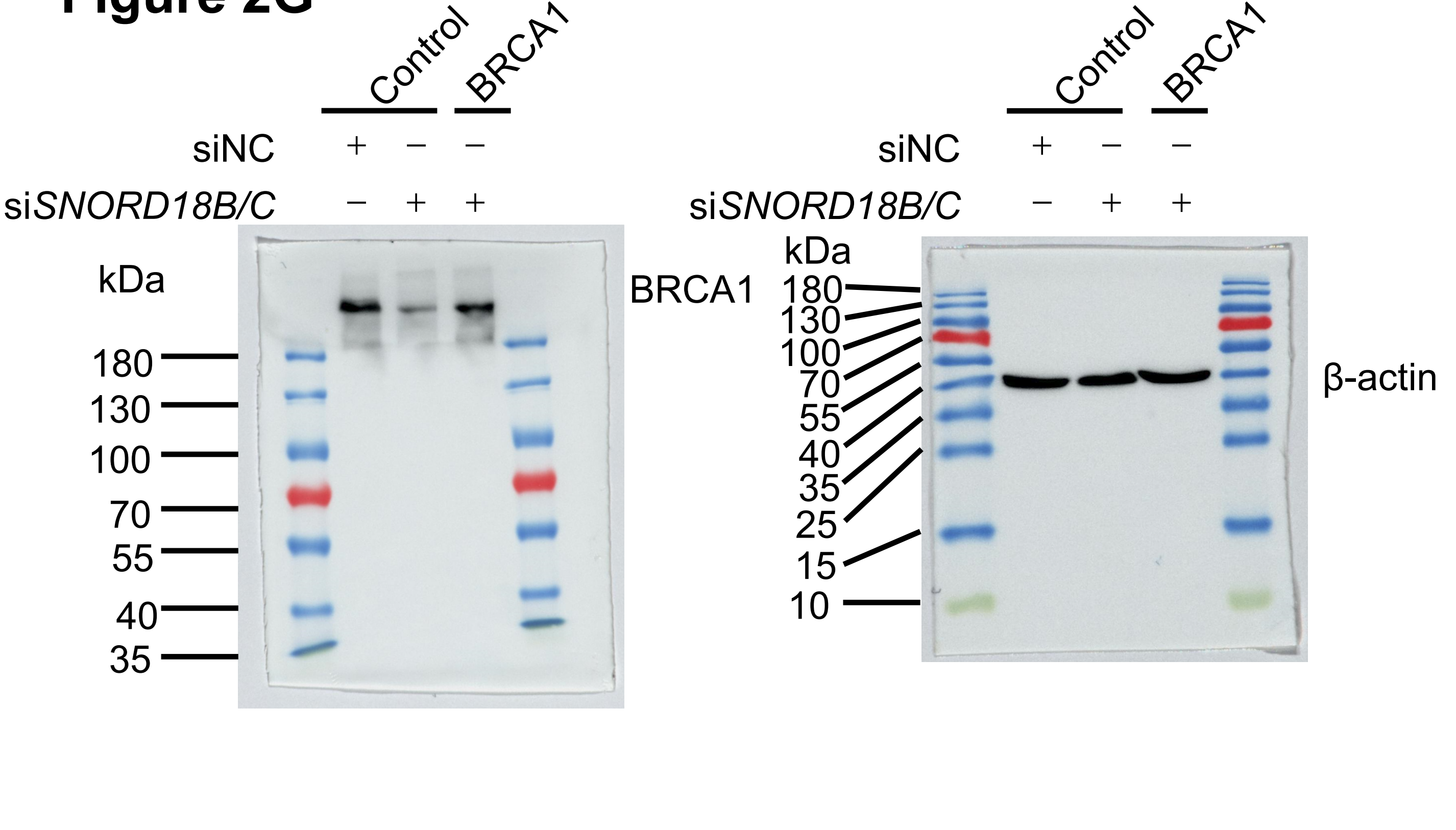

Figure 4E

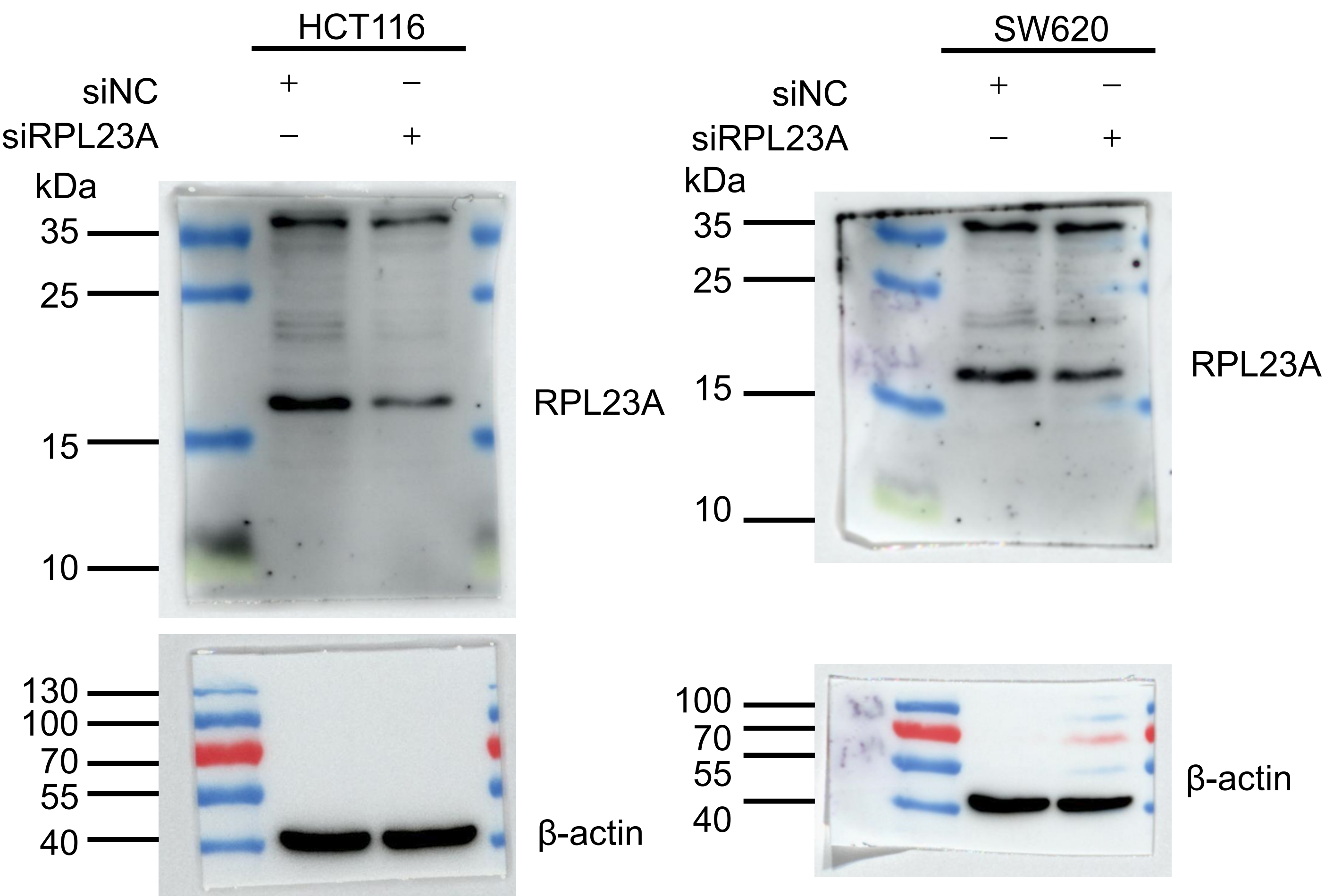
HCT116
siNC + −
siRPL23A − +
kDa
35
25
15
10
RPL23A
130
100
70
55
40
β-actin
SW620
siNC + −
siRPL23A − +
kDa
35
25
15
10
RPL23A
100
70
55
40
β-actin

Figure 4E

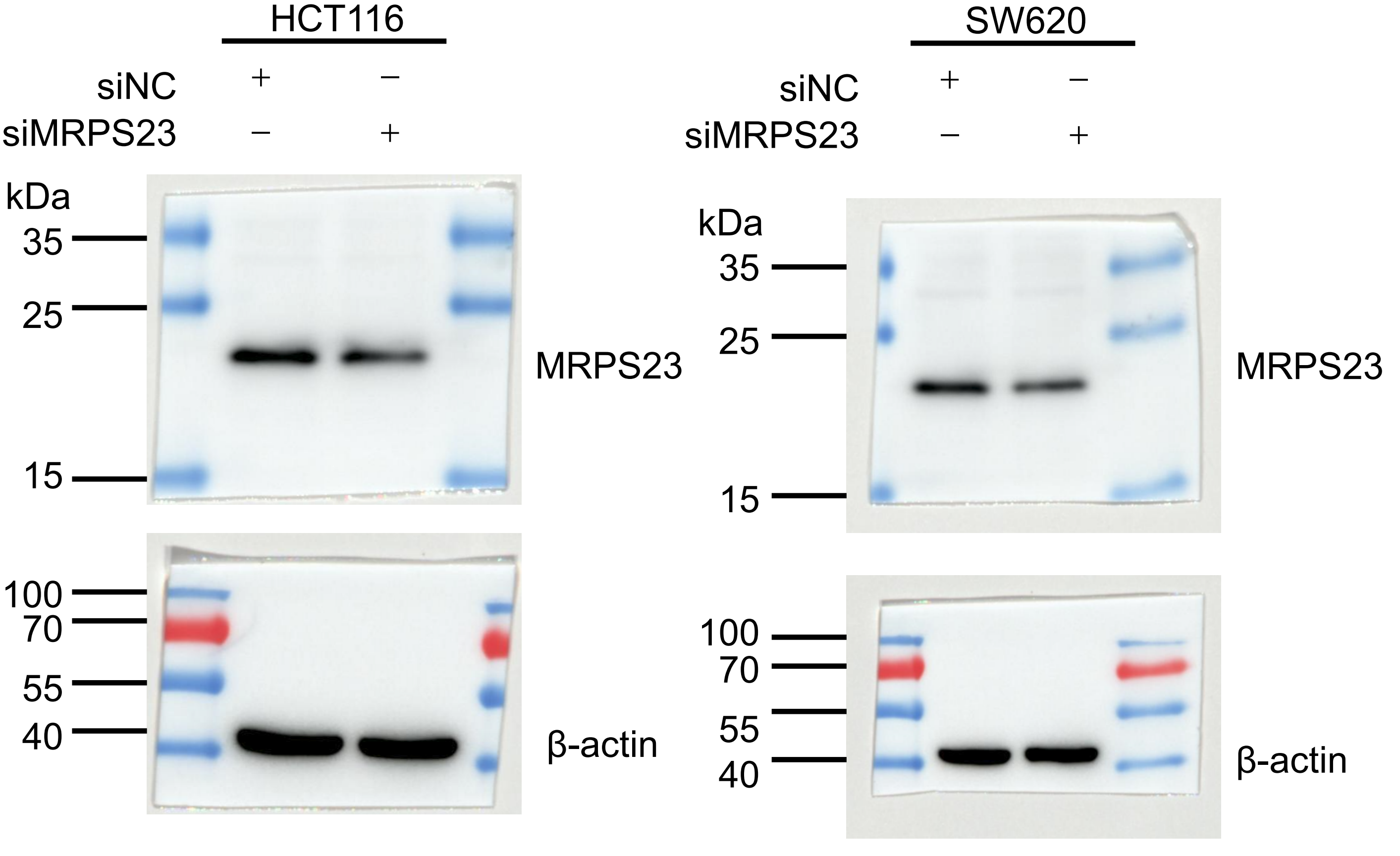

Figure 4G

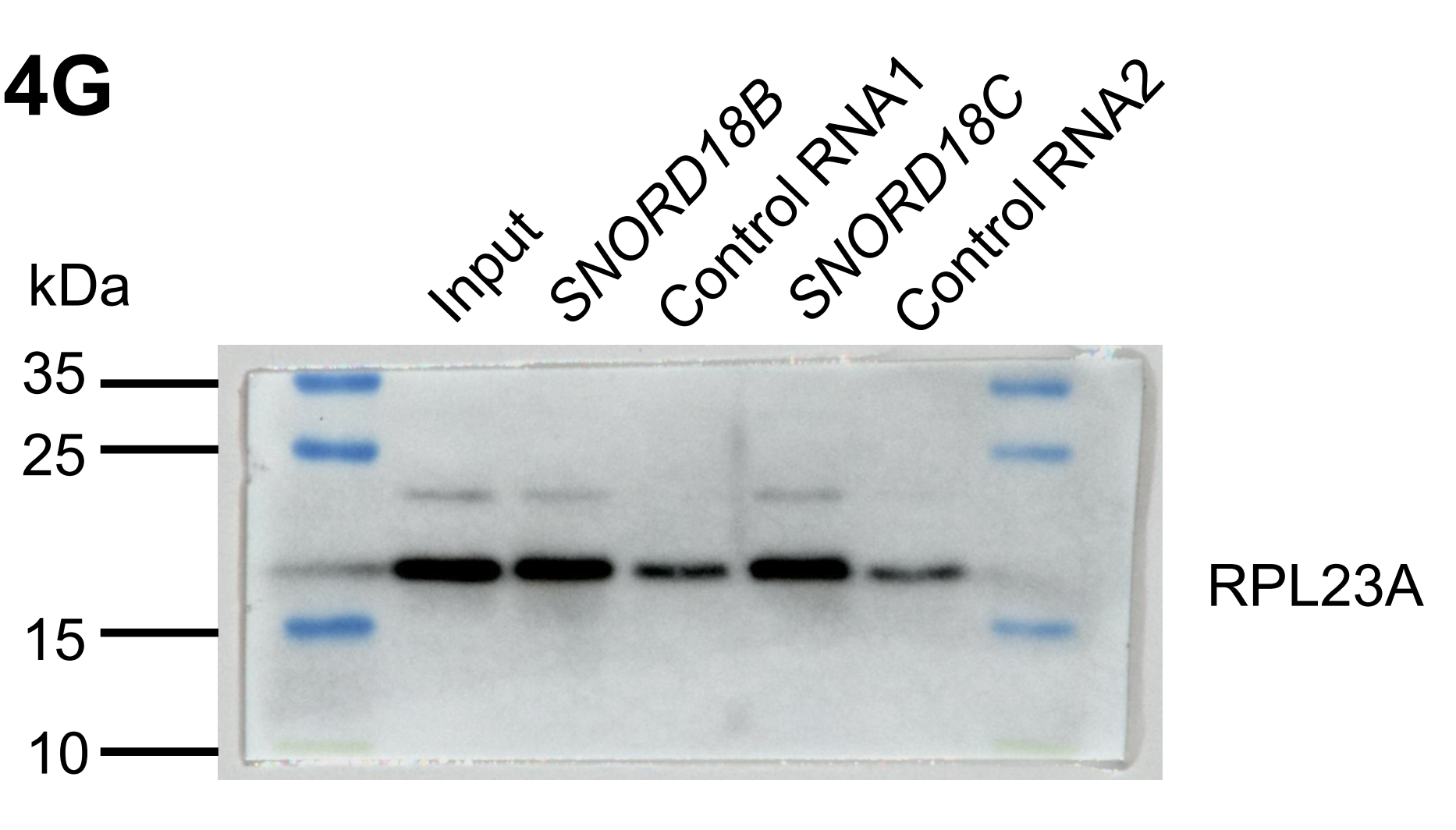

Figure 4I

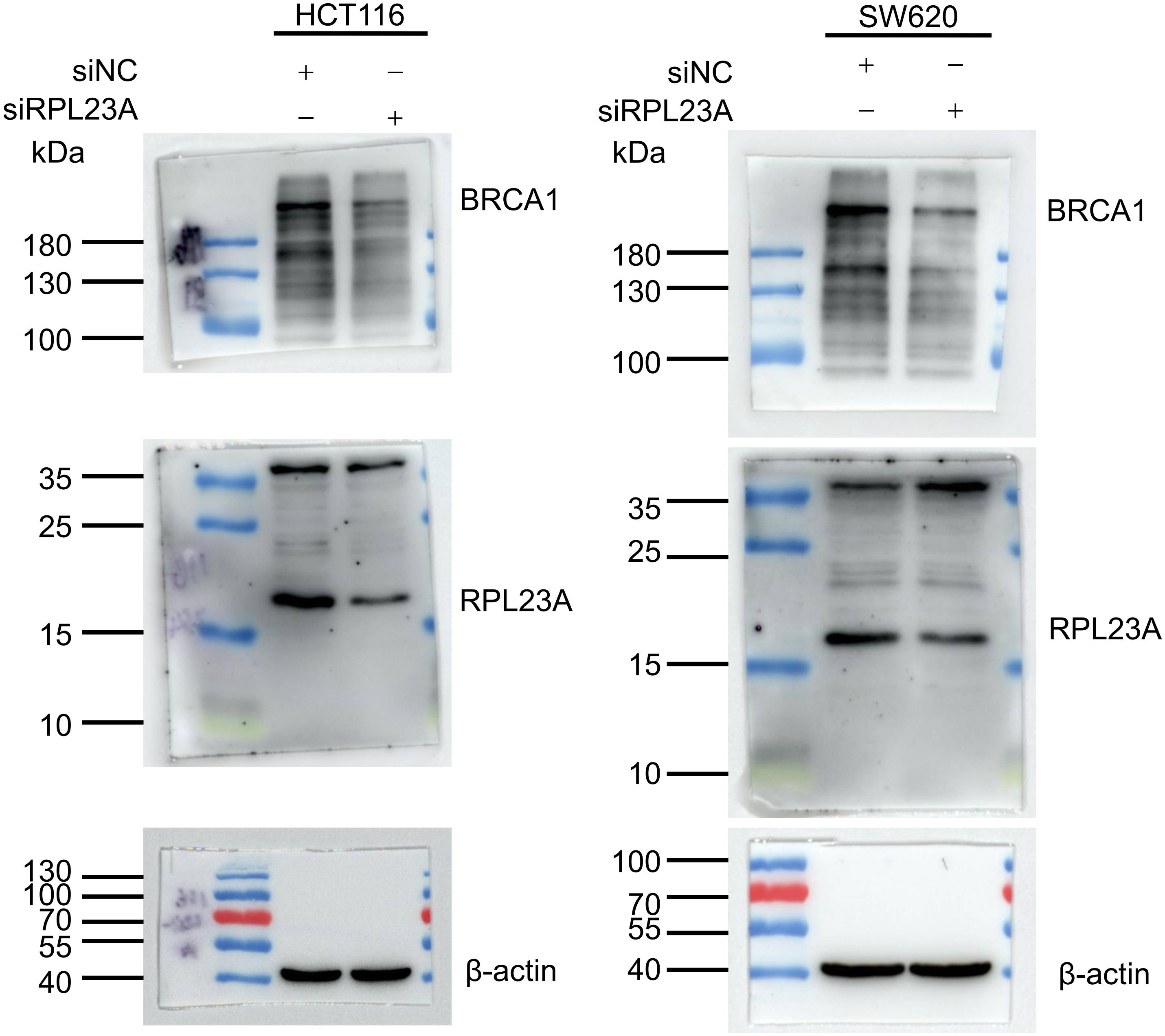
HCT116
siNC
+
–
siRPL23A
–
+
kDa
180
130
100
BRCA1
35
25
15
10
RPL23A
130
100
70
55
40
β-actin
SW620
siNC
+
–
siRPL23A
–
+
kDa
180
130
100
BRCA1
35
25
15
10
RPL23A
100
70
55
40
β-actin

Figure 4K

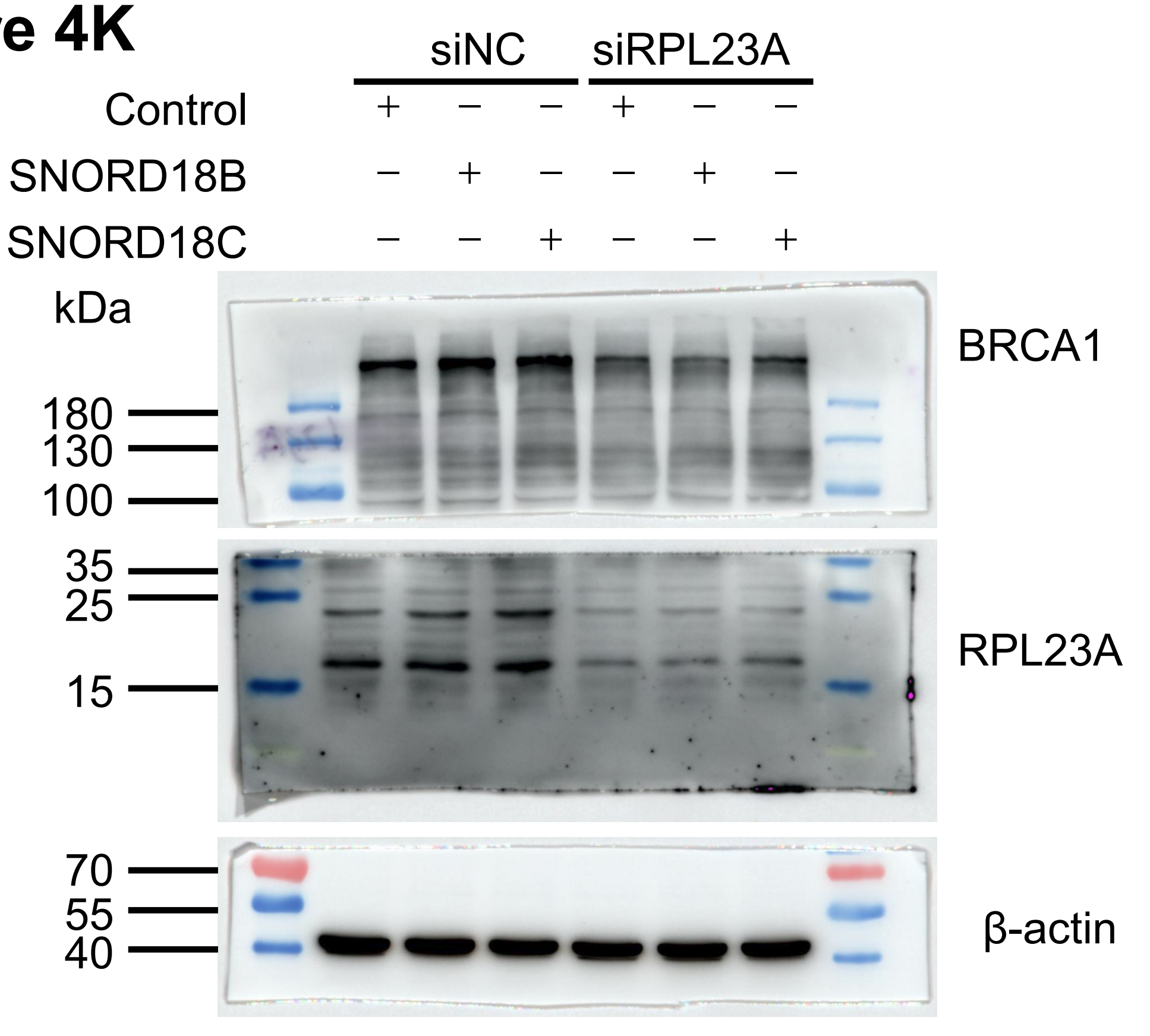

Figure 5A

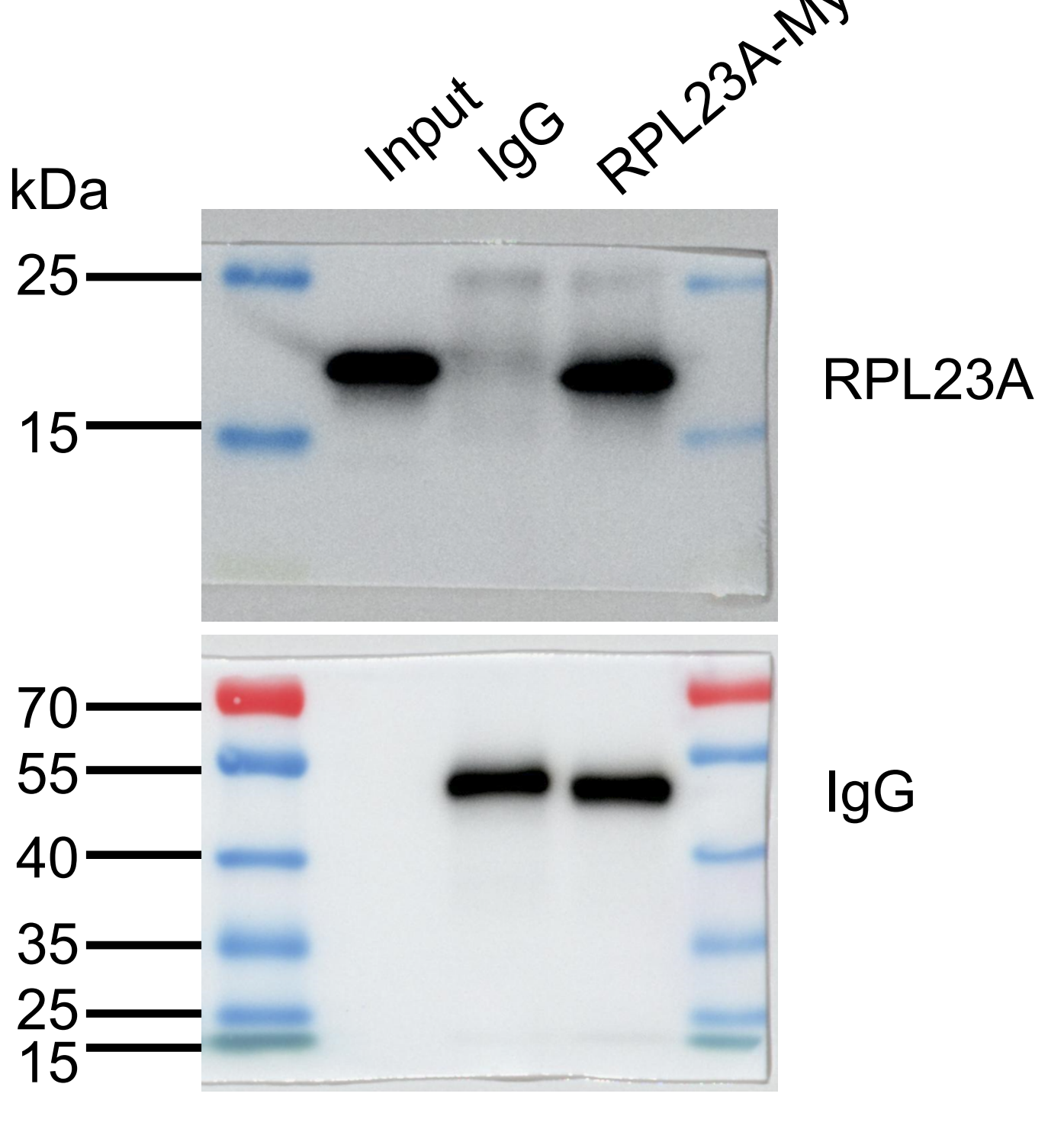

Figure 5B

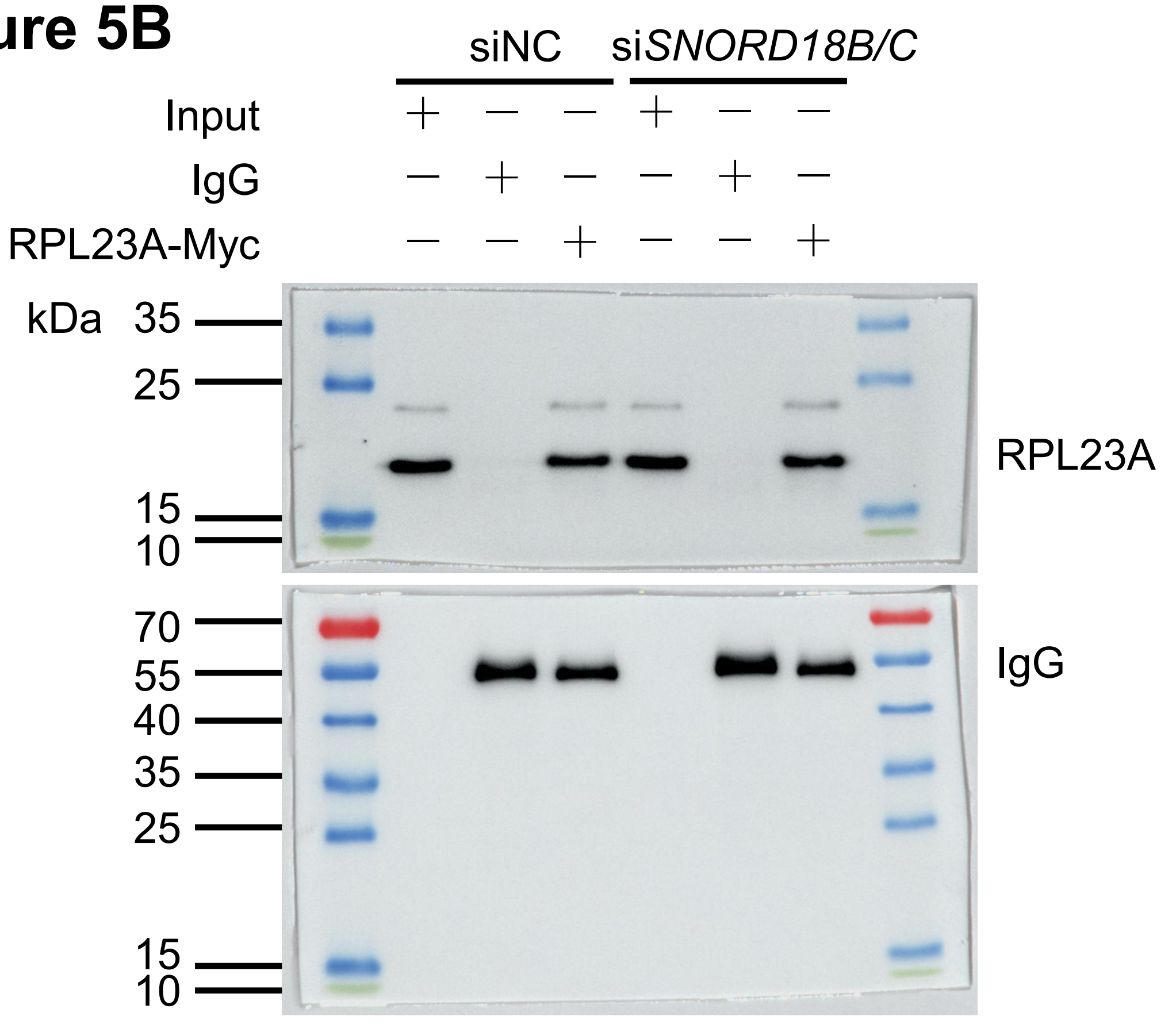

siNC
si*SNORD18B/C*
Input + − − + − −
IgG − + − − + −
RPL23A-Myc − − + − − +
kDa 35
25
15
10
RPL23A
70
55
40
35
25
15
10
IgG

Figure S2C

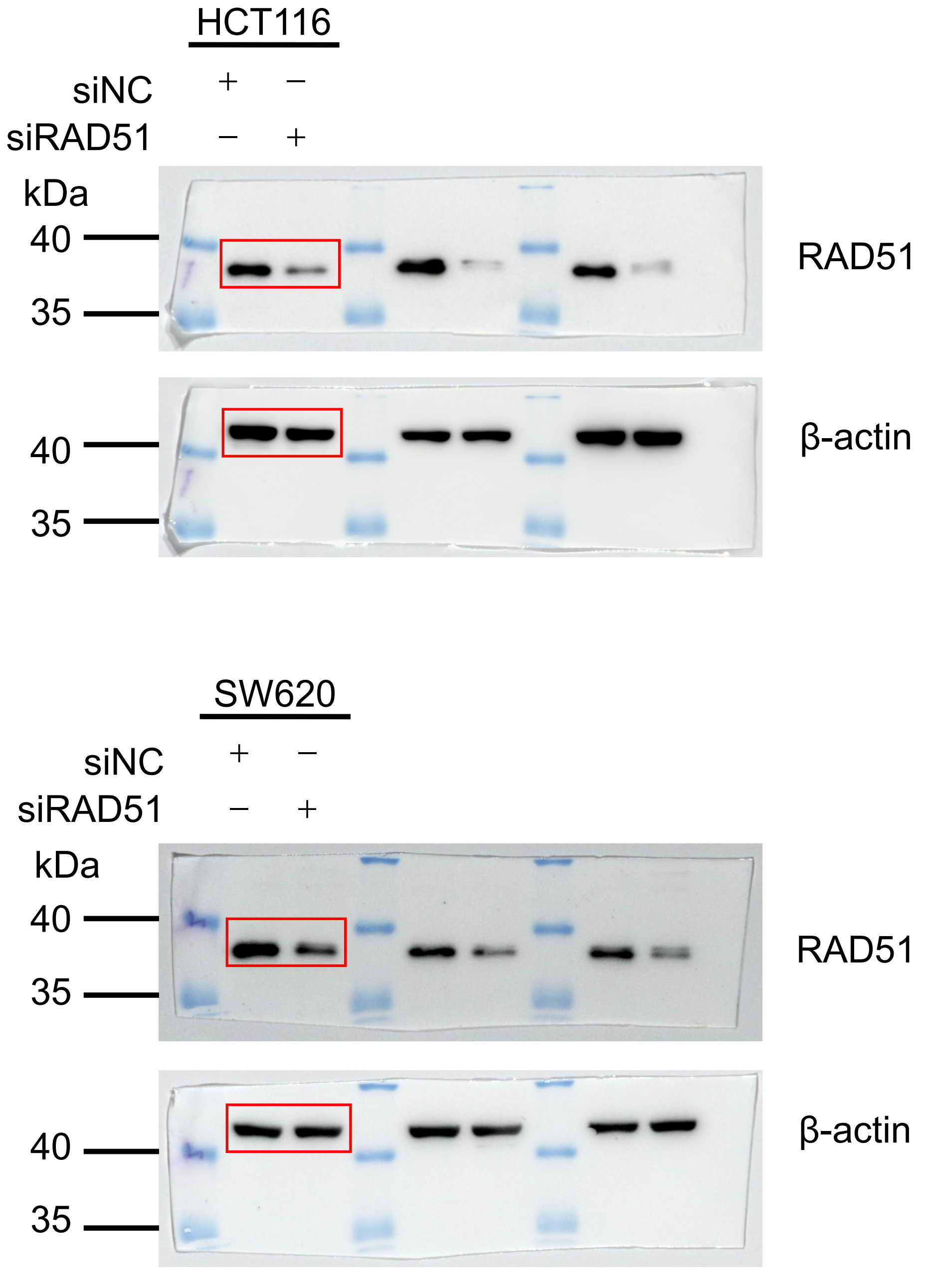
HCT116
siNC + −
siRAD51 − +
kDa
40
35
RAD51
40
35
β-actin
SW620
siNC + −
siRAD51 − +
kDa
40
35
RAD51
40
35
β-actin

Figure S5

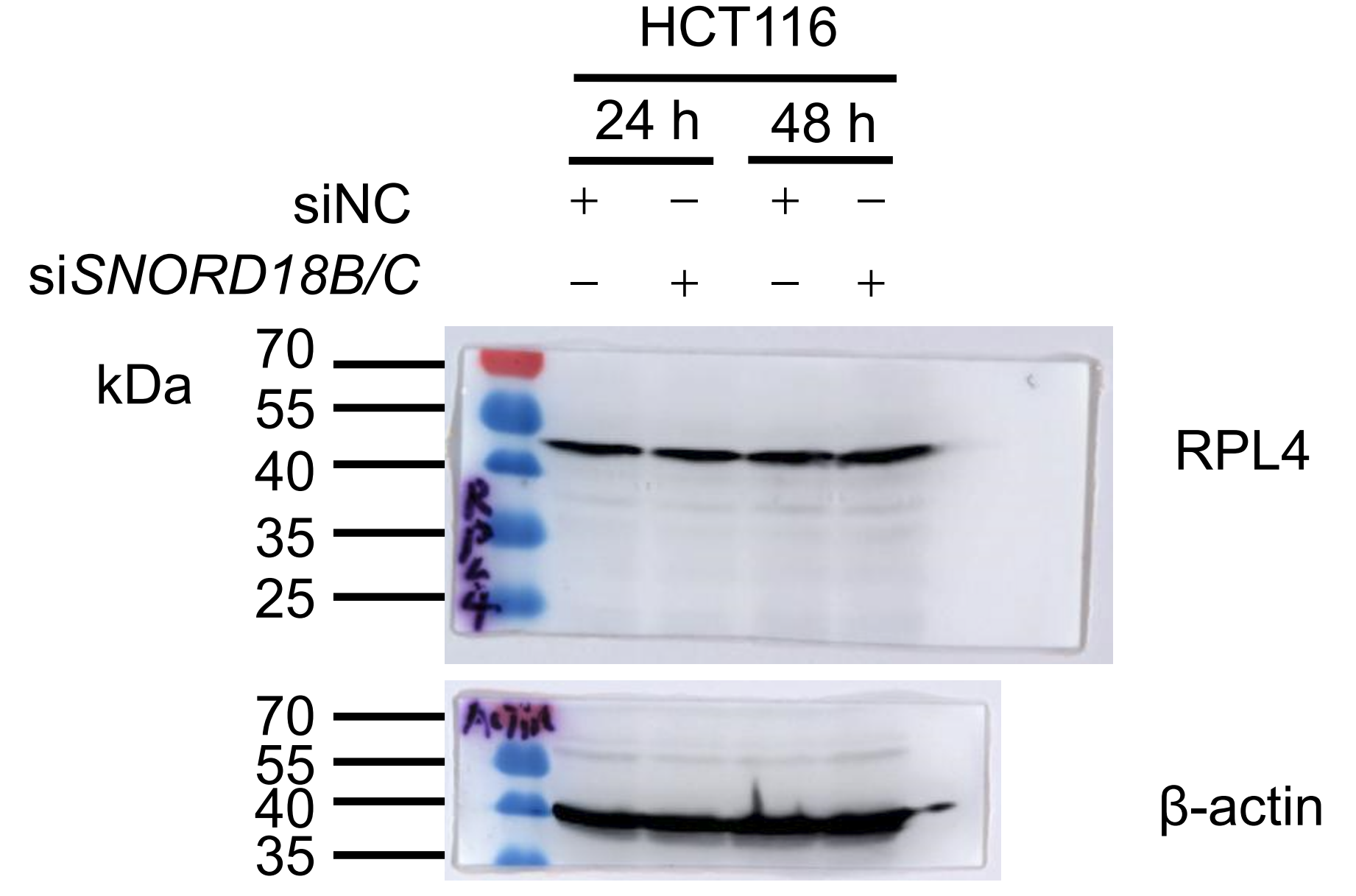
HCT116
24 h
48 h
siNC
+ − + −
si*SNORD18B/C*
− + − +
kDa
70
55
40
35
25
RPL4
70
55
40
35
β-actin

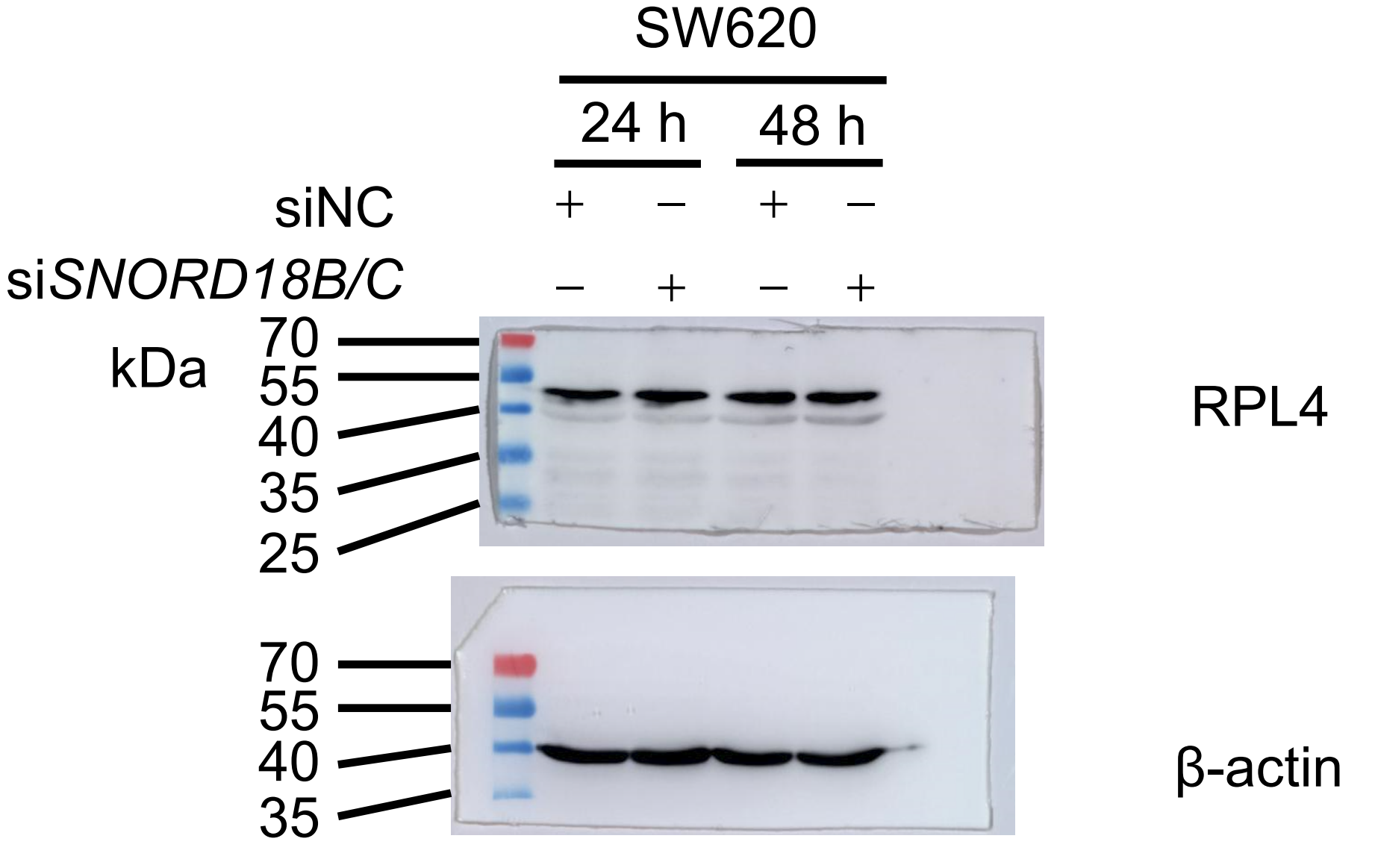
SW620
24 h
48 h
siNC
+ − + −
si*SNORD18B/C*
− + − +
kDa
70
55
40
35
25
RPL4
70
55
40
35
β-actin

Figure S7B

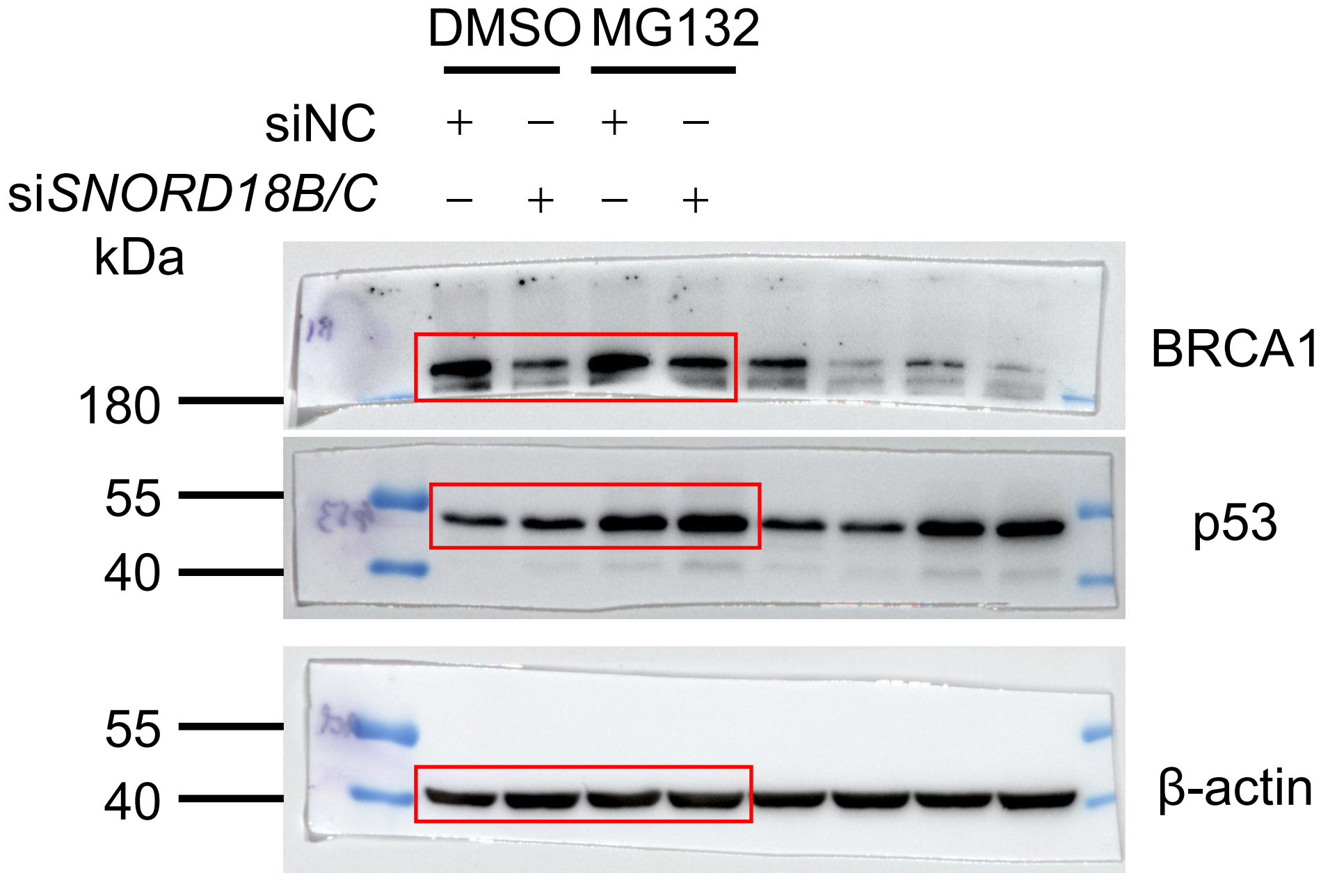

DMSO
MG132
siNC + − + −
si*SNORD18B/C* − + − +
kDa
180
55
40
55
40
BRCA1
p53
β-actin

Figure S7C

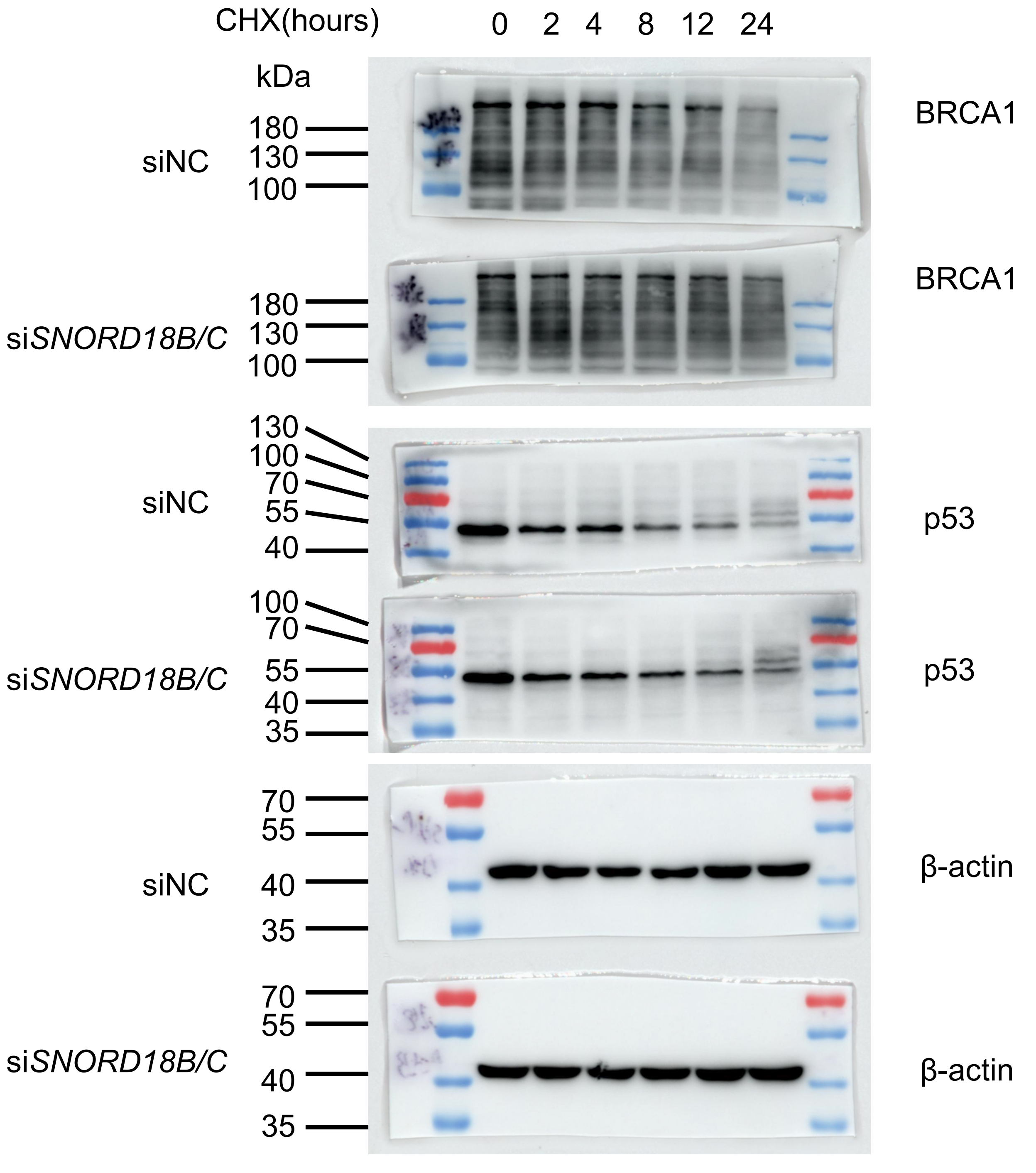
CHX(hours)
0
2
4
8
12
24
kDa
siNC
180
130
100
BRCA1
siSNORD18B/C
180
130
100
BRCA1
siNC
130
100
70
55
40
p53
siSNORD18B/C
100
70
55
40
35
p53
siNC
70
55
40
35
β-actin
siSNORD18B/C
70
55
40
35
β-actin

Figure S8A

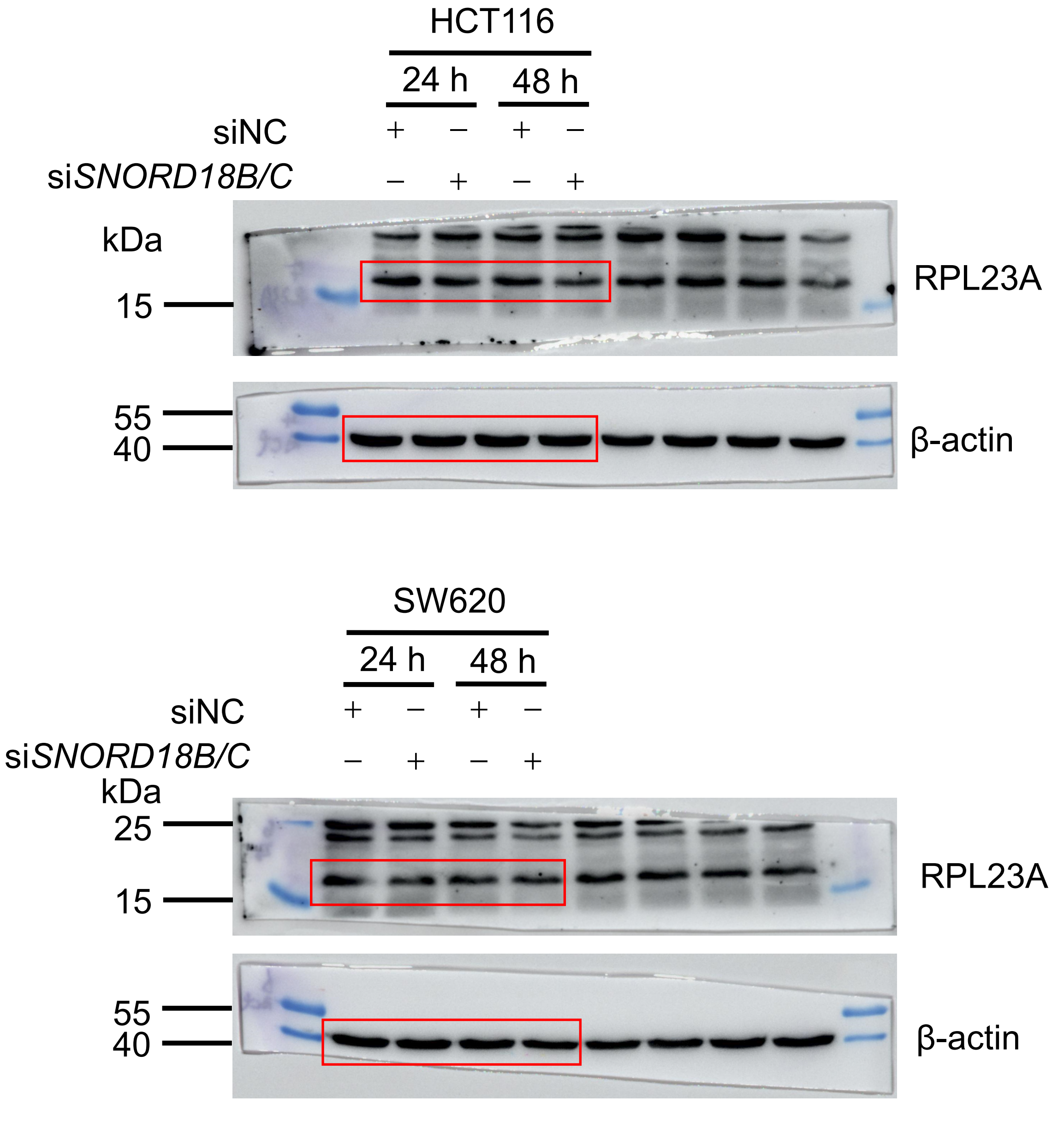
HCT116
24 h
48 h
siNC
siSNORD18B/C
kDa
15
RPL23A
55
40
β-actin
SW620
24 h
48 h
siNC
siSNORD18B/C
kDa
25
15
RPL23A
55
40
β-actin
