# Supplementary material for: SNORD18B/SNORD18C-RPL23A promotes homologous recombination repair by regulating BRCA1 translation in colorectal cancer cells
Source: Cell Death Discov. 2026 Aug 6;12:436. doi: 10.1038/s41420-026-03289-3 (PMC13731662; doi:10.1038/s41420-026-03289-3)
Supplement: Supplementary file 2 [file 41420_2026_3289_MOESM1_ESM.docx]

**Supplementary Information**

***SNORD18B*/*SNORD18C*-RPL23A Promotes Homologous Recombination Repair by Regulating *BRCA1* Translation in Colorectal Cancer Cells** Chuxian Lin^1,#^, Junyan He^2,#^, Lei Wu^1^, Xin Liu^1^, Ying Fan^1^, Dan Cai^3^, Meng Jia^1^, Wencheng Zhang^1^, Qi Wang^1^, Yaqiong Li^1^, Ruixue Liu^1^, Wenqing Lu^1^, Jianan Ma^1^, MaoXiang Zhu^1^, Pingkun Zhou^1^, Zhenhua Qi^1^, Liping Shen^1,*^, Zhidong Wang^1,3,*^

^1^Beijing Institute of Radiation Medicine, Beijing 100850, China.

^2^Department of Oncology, The First Affiliated Hospital, Hengyang Medical School, University of South China, Hengyang, Hunan 421001, China.

^3^Graduate Collaborative Training Base of Academy of Military Sciences, Hengyang Medical School, University of South China, Hengyang, Hunan, 421001, China.

^#^These authors contributed equally to this work.

^*^Correspondence: shen_2011yan@sina.com (L.S.), wangzdlab@126.com (Z.W.)

**Figure S1. *SNORD18B* and *SNORD18C* (*SNORD18B/C*) overexpression in colorectal cancer and radiation-exposed mouse models.**

**A** Left: Heatmap displaying top 12 upregulated snoRNAs (FC >1.3, irradiated versus control) in mouse peripheral blood lymphocytes after ^60^Co γ-ray exposure. Right: Intersection of two CRC-upregulated snoRNA across two independent cohorts: Cohort 1 (GEO accession: GSE267087) and Cohort 2 (DOI: 10.1155/2020/8284576). **B** The SNORD18B/C expression in the mentioned models. **C** Homology analysis of *SNORD18B* and *SNORD18C*. *SNORD18B* and *SNORD18C* sequences were aligned using the Needleman-Wunsch algorithm through NCBI BLAST. **D** Genomic localization mapping of SNORD18B/C within host gene RPL4 (*Homo sapiens* genome).

**Figure S2.** **Expression profiling of *SNORD18B/C* and RAD51 in HCT116 and SW620 cell lines.**

**A** HCT116 and SW620 cells were transfected with 80 nM control siRNA or *SNORD18B/C*-targeting siRNAs, followed by qRT-PCR analysis 48 hours post-transfection. **B** Lentiviral infection (10 MOI) with LV-*NC*, LV-*SNORD18B*, or LV-*SNORD18C* was performed in HCT116 and SW620 cells, with qRT-PCR analysis conducted 72 hours post-infection (two-tailed Student’s *t*-test, *n* = 3). **C** HCT116 and SW620 cells were transfected with 80 nM control siRNA or *RAD51*-targeting siRNAs, followed by Western blot analysis 48 hours post-transfection. **D** qRT-PCR quantification of gene expression in HCT116 cells containing wild-type *SNORD18B/C* or specific mutations (*SNORD18B* or *SNORD18C*) (two-tailed Student’s *t*-test, *n* = 3). ****P<0.001*

**Figure S3. Effect of *SNORD18B/C* on NHEJ repair.** HCT116 and SW620 cell lines with *SNORD18B/C* knockdown (**A**) or overexpression (**B**) were co-transfected with linearized EJ5-GFP and p-Cherry plasmids. The proportion of GFP^+^ cells was quantified 24 hours post-transfection using a two-tailed Student’s *t*-test (*n* = 3, mean ± SD). **P*<0.05; ***P*<0.01, *ns* means no significance

**Figure S4. *SNORD18B/C* enhances irradiation-induced RAD51 foci formation in SW620.** RAD51 immunofluorescence was analyzed in SW620 cells with *SNORD18B/C* knockdown (**A**) or overexpression (**B**) at 2 and 4 hours post 10 Gy irradiation (scale bar: 5 μm). Foci numbers in ≥200 nuclei per group were quantified using Fiji software (Mann‒Whitney U test). **P*<0.05, ***P*<0.01, ****P*<0.001

**Figure S5. *SNORD18B/C* knockdown does not alter the expression of RPL4.** HCT116 and SW620 cells were transfected with 80 nM negative control siRNA or *SNORD18B/C*-targeting siRNAs. Western blot analysis was performed at 24 and 48 hours post-transfection.

**Figure S6. The impact of *SNORD18B/C* overexpression on the** **cell cycle.** The cell cycle distributions of HCT116 and SW620 cells stably infected with LV-NC, LV-*SNORD18B*, and LV-*SNORD18C* were analyzed by flow cytometry following 48 hours culture. Two-tailed Student’s *t*-test, *n* = 3. **P*<0.05; ***P*<0.01, ****P*<0.001, *ns* means no significance

**Figure S7. Analysis of *SNORD18B/C*-mediated regulation of BRCA1.**

**A** *SNORD18B/C* knockdown does not alter BRCA1 mRNA. HCT116 and SW620 cells were analyzed by qRT-PCR at 48 hours post-transfection with *SNORD18B/C*-specific siRNAs. BRCA1 mRNA expression levels were normalized to *ACTB* (two-tailed Student’s *t*-test, *n* = 3, *ns* means no significance). **B-C** *SNORD18B/C* depletion does not alter BRCA1 protein stability. (**B**) HCT116 cells transfected with si*SNORD18B/C* or siNC were treated with DMSO or 10 μM MG132 for 12 hours. (**C**) Control and SNORD18B/C-depleted HCT116 cells were exposed to 100 μg mL^-1^ cycloheximide or DMSO for 0, 2, 4, 8, 12, 24 hours. Residual BRCA1 and p53 protein levels were quantified by western blotting. *ns* means no significance

**Figure S8. Expression independence between *SNORD18B/C* and RPL23A in CRC cells.**

**A** *SNORD18B/C* knockdown does not modulate RPL23A protein levels. Western blot analysis of RPL23A expression in HCT116 and SW620 cells following *SNORD18B/C*-specific siRNA transfection at 24 and 48 hours. **B** RPL23A knockdown does not regulate *SNORD18B/C* expression. qRT-PCR analysis of *SNORD18B/C* levels in HCT116 and SW620 cells after 48 hour RPL23A siRNA transfection (two-tailed Student’s *t*-test, *n* = 3). **P*<0.05, ***P*<0.01, *ns* means no significance

**Figure S9. *SNORD18B/C* promoted CRC cell proliferation.**

Cell proliferation analysis of HCT116 and SW620 cells with *SNORD18B/C* knockdown (**a**), knockout (**b**), and upregulation (**c**). HCT116 and SW620 cells were seeded in a 96-well plate (1 000 cells per well) and subjected to CCK-8 assays at 0, 24, 48 and 72 hours (two-tailed Student’s *t*-test, *n* = 5). ****P*<0.001

**Figure S10. Increased expression of *SNORD18B/C* inhibited DNA damage-induced apoptosis.**

Apoptosis in HCT116 cells lines (control, *SNORD18B*, and *SNORD18C*) was detected (**A**) post-10 Gy irradiation (48 hours) or (**B**) treated with 400 μM H_2_O_2_ (8 hours) (Mean ± SD, two-tailed Student’s *t*-test, *n* = 3). **P*<0.05; ***P*<0.01, *ns* means no significance

**Figure S11.** ***SNORD18B/C* enhances CRC xenograft radioresistance *in vivo*.**

Xenograft models were established using HCT116 cells with *SNORD18B or SNORD18C* knockout or overexpression. (**A, E)** Representative images of xenograft-bearing mice at the experimental endpoint. **(B, F)** Final tumor weights. **(C, G)** *SNORD18B/C* expression levels in xenograft tumors post-irradiation. **(D, H)** H&E staining and Ki67 IHC analyses of xenograft tumors post-irradiation (scale bar = 100 μm). (two-tailed Student’s *t*-test, *n* = 6). **P*<0.05, ***P*<0.01, *** *P*<0.001, *ns* means no significance
